# Suprapontine Structures Modulate Brainstem and Spinal Networks

**DOI:** 10.1007/s10571-023-01321-z

**Published:** 2023-02-02

**Authors:** Atiyeh Mohammadshirazi, Rosamaria Apicella, Benjamín A. Zylberberg, Graciela L. Mazzone, Giuliano Taccola

**Affiliations:** 1 Neuroscience Department, International School for Advanced Studies (SISSA), Via Bonomea 265, 34136 Trieste, Italy; 2 Applied Neurophysiology and Neuropharmacology Lab, Istituto di Medicina Fisica e Riabilitazione (IMFR), Via Gervasutta 48, Udine, UD Italy; 3 Instituto de Investigaciones en Medicina Traslacional (IIMT)-CONICET - Universidad Austral, Av. Pte. Perón 1500, Pilar, Buenos Aires, Argentina

**Keywords:** Motor-evoked potentials, Fictive respiration, Fictive locomotion, Decerebration, Leg attached, Isolated central nervous system, Tissue oxygenation

## Abstract

Several spinal motor output and essential rhythmic behaviors are controlled by supraspinal structures, although their contribution to neuronal networks for respiration and locomotion at birth still requires better characterization. As preparations of isolated brainstem and spinal networks only focus on local circuitry, we introduced the in vitro central nervous system (CNS) from neonatal rodents to simultaneously record a stable respiratory rhythm from both cervical and lumbar ventral roots (VRs).

Electrical pulses supplied to multiple sites of brainstem evoked distinct VR responses with staggered onset in the rostro-caudal direction. Stimulation of ventrolateral medulla (VLM) resulted in higher events from homolateral VRs. Stimulating a lumbar dorsal root (DR) elicited responses even from cervical VRs, albeit small and delayed, confirming functional ascending pathways. Oximetric assessments detected optimal oxygen levels on brainstem and cortical surfaces, and histological analysis of internal brain structures indicated preserved neuron viability without astrogliosis. Serial ablations showed precollicular decerebration reducing respiratory burst duration and frequency and diminishing the area of lumbar DR and VR potentials elicited by DR stimulation, while pontobulbar transection increased the frequency and duration of respiratory bursts. Keeping legs attached allows for expressing a respiratory rhythm during hindlimb stimulation. Trains of pulses evoked episodes of fictive locomotion (FL) when delivered to VLM or to a DR, the latter with a slightly better FL than in isolated cords.

In summary, suprapontine centers regulate spontaneous respiratory rhythms, as well as electrically evoked reflexes and spinal network activity. The current approach contributes to clarifying modulatory brain influences on the brainstem and spinal microcircuits during development.

**Graphical Abstract:**

Novel preparation of the entire isolated CNS from newborn rats unveils suprapontine modulation on brainstem and spinal networks. Preparation views (A) with and without legs attached (B). Successful fictive respiration occurs with fast dissection from P0-P2 rats (C). Decerebration speeds up respiratory rhythm (D) and reduces spinal reflexes derived from both ventral and dorsal lumbar roots (E).

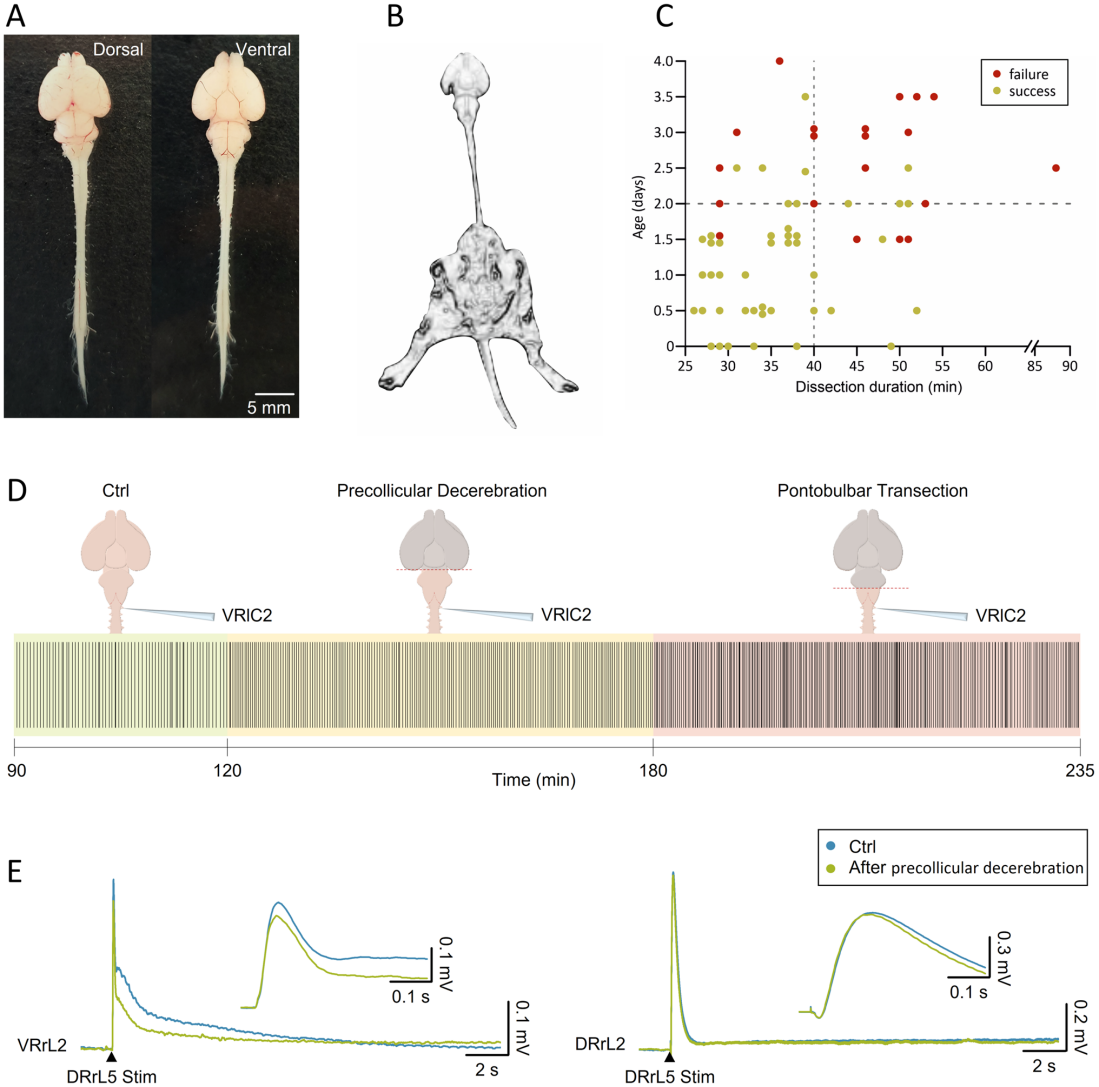

## Introduction

Locomotion is a complex motor behavior resulting from the continuous integration of multiple neuronal input. Descending commands from the brain trigger and modulate the intrinsic rhythmic activity of spinal circuits, which are further refined by continuous afferent sensory signals from the periphery. Disconnection from higher centers and the complete deafferentation from the body periphery make the neonatal rodents’ spinal cord, isolated from the lower thoracic segments to the cauda equina, an optimal model to address both development and functional organization of the rhythmogenic lumbar networks (named central pattern generators, CPGs) responsible for generating the patterned activation of lower limb muscles during locomotion (Kiehn and Butt 2003). Indeed, the pharmacological modulation of locomotor CPGs (Cazalets et al. 1992; Blivis et al. 2007; Tazerart et al. 2008; Dose et al. 2014) and their recruitment by repetitive electrical stimulation of dorsal afferents (Marchetti et al. 2001; Etlin et al. 2010; Taccola 2011; Dose and Taccola 2016) have been successfully described using the isolated spinal cord from neonatal rodents. Furthermore, preparations of the isolated spinal cord with legs attached were introduced both for tracing real stepping and muscle recruitment during ongoing CPG activation (Kiehn and Kjaerulff 1996) and for eliciting afferent input aimed at spinal networks (Mandadi and Whelan 2009; Dingu et al. 2018).

Noteworthy, these reduced preparations focus on local spinal microcircuits. A more conservative approach also considers the presence of intact pons and brainstem (Suzue 1984) to explore the descending activation of lumbar circuits through repetitive electrical pulses applied to the ventrolateral medulla (VLM; Zaporozhets et al. 2004) and to investigate the functional coupling between locomotor spinal circuits and respiratory networks located in the brainstem (Giraudin et al. 2012). However, all these reduced preparations preclude the possibility to explore the modulatory role of suprapontine structures on spinal and brainstem circuits at birth, as well as the changes in these interactions during development.

Compelling evidence from in vivo animals shows suprapontine structures modulating respiration and locomotion already in newborns (Horn and Waldrop 1998). Still in newborns, it has been demonstrated that the caudal hypothalamus affects respiratory function (Lakke 1997; Dreshaj et al. 2003), and basal ganglia are pivotal for regulating postural muscle tone during rhythmic motor behavior of limbs (Van Hartesveldt and Lindquist 1978; Takakusaki et al. 2004). However, an in vitro preparation of the entire rodent CNS has never been introduced, as it is widely accepted that the postnatal rodent tissue likely suffers from hypoxia if the dissection lasts longer than the brief time required for isolating the sole spinal cord (Wilson et al. 2003). The pioneering work of John Nicholls tried to circumvent this limitation by introducing a more immature preparation using opossums at birth (Nicholls et al. 1990), corresponding to 14-day rat embryos, as they are less vulnerable to hypoxic conditions. The whole CNS isolated in vitro from opossum neonates allowed to acquire several important outcomes, such as compound action potentials evoked by stimulating the CNS, spinal reflexes, and the spontaneous rhythmical nerve activity related to respiration. Histological viability of the entire CNS isolated from newborn opossums was confirmed for up to four days in Krebs’ fluid, and up to seven in enriched media under sterile conditions (Nicholls et al. 1990; Eugenín and Nicholls, 2000). However, due to its immaturity, the postnatal opossum preparation lacks the cerebellum, depriving the study of its potential contribution to modulating respiratory and motor functions (Lutherer et al. 1989).

The importance of exploring suprapontine influences on both brainstem and spinal networks in a more structured mammalian preparation of the entire CNS in vitro, made us wonder whether well-established faster procedures of tissue dissection on 0–2-day-old pups only, would allow isolation of the complete CNS, cerebellum included, from neonatal rats.

The functionality of the preparation was assessed by: the persistence of a spontaneous respiratory rhythm from cervical roots for at least 4 hours from anesthesia, the well-preserved oxygen levels in cortical and brainstem tissues for the entire length of experiments, the histological validation of neuronal viability after 4 hours in vitro, the presence of electrically evoked motor responses after brainstem and spinal cord stimulation, and the expression of episodes of locomotor-like oscillations elicited by trains of pulses delivered to dorsal afferents and VLM. All of the tested functional outcomes were subject to some modulatory influences from suprapontine centers.

In summary, the current study defined an in vitro preparation of the entire mammalian CNS for studying the cellular basis of rhythmical activity and their development in the first postnatal days. In addition, the novel experimental setting clarified the presence, from the first days of life, of modulatory influences from suprapontine structures, which determine faster and broader respiratory bursts, while also marginally facilitating episodes of locomotor-like cycles elicited by afferent stimulation.

## Methods

### In Vitro Preparation of the Isolated Entire CNS

All procedures were approved by the International School for Advanced Studies (SISSA) ethics committee and are in accordance with the guidelines of the National Institutes of Health (NIH) and with the Italian Animal Welfare Act 24/3/2014 n. 26, implementing the European Union directive on animal experimentation (2010/63/EU). All efforts were made to minimize the number and suffering of animals. A total of 148 postnatal Wistar rats (P0–P4) of both sexes were used at random.

**Fig. 1:**
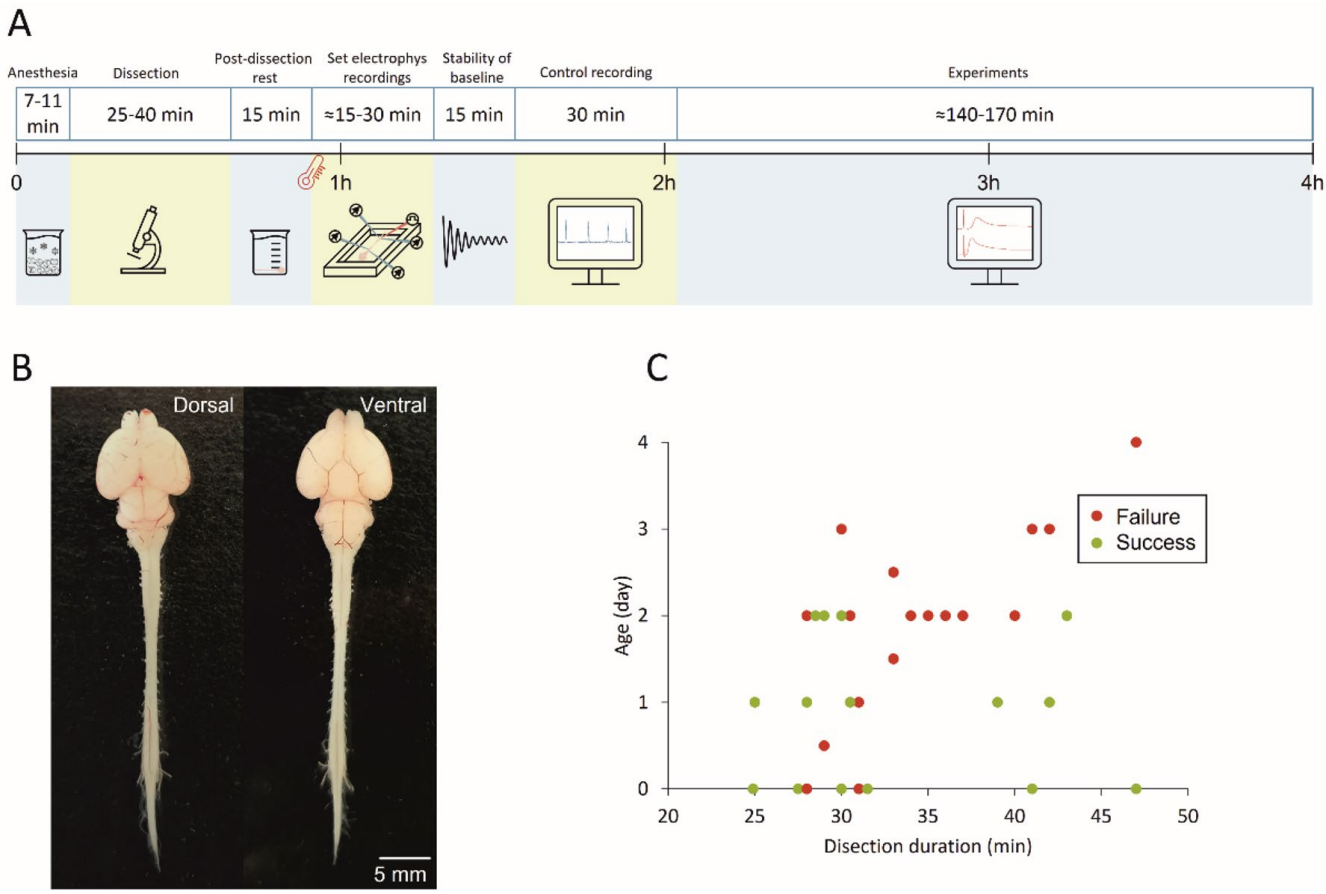
Experimental design of the study. **A** A timeline describing the experimental procedures, from cryoanesthesia to dissection, post-dissection rest, setting of electrophysiological recordings, pause to stabilize the baseline, and, eventually, control and experimental recordings. Note that as soon as the preparation was placed in the recording chamber, bath temperature was progressively raised to 25–27 °C. **B** Photographs displaying dorsal and ventral views of the isolated entire CNS preparation from a P1 newborn. **C** Scatter plot describes the presence of a stable fictive respiration for each preparation, arranged by age and time of dissection (*n =* 32). Green dots correspond to successes (> 4 h of fictive respiration), while red dots represent failures (< 4 h of fictive respiration)

As graphically summarized by the timeline in Fig. 1A, 7–11 minutes of cryoanesthesia (Phifer and Terry 1986; Danneman and Mandrell 1997; Zimmer et al. 2020) anticipated surgical procedures at room temperature. After the disappearance of the tail pinch reflex, the forehead was ablated at the level of the orbital line, the skin removed from the animal’s skull and back, and the chest and forelimbs ventrally detached. The preparation was then placed on a Sylgard-filled petri dish under a microscope and fully covered with oxygenated Krebs solution, which was frequently replaced. Krebs solution contained (in mM): 113 NaCl, 4.5 KCl, 1 MgCl_2_7H_2_O, 2 CaCl_2_, 1 NaH_2_PO_4_, 25 NaHCO_3_, and 30 glucose, gassed with 95% O_2_-5% CO_2_, pH 7.4, 298 mOsm/kg. Afterward, craniotomy and ventral and dorsal laminectomies were performed to expose the entire CNS, which was then isolated from the olfactory bulbs down to the cauda equina by carefully transecting all cranial nerves, dorsal roots (DRs) and ventral roots (VRs; Nicholls et al. 1990). Figure 1B pictures the whole CNS preparation from a P1 newborn in dorsal and ventral views. On average, dissection procedures lasted about 30 min. A post-dissection resting period of 15 min was systematically respected after surgical dissection (Fig. 1A).

For electrophysiological recordings, the preparation was then placed ventral side up in a recording chamber (Fig. 1A; chamber depth = 4000 µm) continuously perfused with Krebs solution (7 mL/min), while the bath temperature was progressively raised and maintained in the range of 25 to 27 °C by a single channel temperature controller (TC-324C Warner Instruments, USA). Once the baseline stabilized (15 min; Fig. 1A), 30 min of stable control (Fig. 1A) was acquired through extracellular recordings.

For the preparation of the entire CNS with legs attached, dorsal and ventral laminectomies were performed down to the lowest thoracic level (Th13), preserving the remaining lumbosacral vertebra and nerves attached to hindlimbs. DRs and VRs were kept as long as possible, removing only the dorsal root ganglia (DRG).

Plots in Figs. 1C and 9B report the age of the animal on the x-axis and the length of surgical dissection on the y-axis, identifying a bottom-left region (younger preparations undergoing fast surgical procedures) where preparations showed the highest percentage of successfully recorded respiratory rhythms for at least 4 h from the anesthesia performed at the beginning of tissue isolation. Based on the outcome of this set of preliminary experiments, P0-3 newborns were selected for the rest of the study.

### Extracellular Recordings

DC-coupled recordings were acquired from VRs and DRs through tight-fitting suction electrodes connected to a differential amplifier (DP-304, Warner Instruments, Hamden, CT, USA; high-pass filter = 0.1 Hz, low-pass filter = 10 kHz, gain × 1000), then digitized (Digidata 1440, Molecular Devices Corporation, Downingtown, PA, USA; digital Bessel low-pass filter at 10 Hz; sampling rate = 50 kHz) and visualized real-time with the software Clampex 10.7 (Molecular Devices Corporation, Downingtown, PA, USA).

### Electrical Stimulation

Single and repetitive rectangular electrical pulses (duration* =* 0.1 ms, frequency = 0.03 Hz) were delivered to caudal DRs (L4-S1) through a programmable stimulator (STG4002, Multichannel System, Reutlingen, Germany) using bipolar glass suction electrodes with two close silver wires spaced by 300–500 µm. The intensity of stimulation (6–800 µA) is expressed as times to threshold, where the latter is the lowest intensity supplied to a DR to elicit an appreciable depolarizing potential from the homolateral VR. As for input–output experiments, to elicit motor potentials in response to DR stimulation (DRVRPs), 30 single pulses (duration* =* 0.1 ms) at intensities of 1, 1.5, 2, 3, 5 × Thr were delivered at a frequency of 0.03 Hz.

Punctiform stimulation of multiple sites of the brain was conducted with a custom-made bipolar concentric electrode composed of an internal 250-µm-width stainless steel electrode (UE KK1, FHC, Bowdoinham, USA) and a helical silver wire wrapped around the tip of a glass pipette (700-µm-diameter tip). Multisite stimulation of the brainstem consists in a train of 30 rectangular pulses at 0.03 Hz (duration* =* 1 ms; intensity = 2 × Thr, 1800–5000 µA). In experiments involving brainstem stimulation, threshold was defined as the lowest intensity applied to the VLM to obtain an appreciable depolarizing response from lower lumbar VRs. In each experiment, 10 distinct stimulating spots (named A–J) were consistently found on each side of the brainstem based on the anatomy of visible ventral arteries. Two stimulating sites are positioned on the median basal artery (namely A on the most rostral pons, and G at the intersection of basilar and anterior inferior cerebellar arteries). B is positioned about 0.5 mm laterally from A. The spots named C, D and E are aligned on the pons, equally interspaced by 0.5 mm and span from the median basal artery (C) to the lateral extremity of the ventral brainstem (E). In the rostral caudal direction, D is equidistant between superior and inferior cerebellar arteries. The F-stimulating spot is placed on the anterior inferior cerebellar artery between the median basal artery and the lateral ventral edge of the brainstem. H and I are aligned on the medulla, proximal (H) and distal (I) to the median basal artery. In the rostral caudal direction, they are equidistant from the inferior cerebellar artery and the first cervical VR. J is located on the first cervical segment of the spinal cord, laterally to the ventral spinal artery.

In a subgroup of preparations, the stimulation site was visually confirmed at the end of the experiment by electrolytically destroying the area of stimulation through strong electrical pulses (intensity = 16 mA, duration* =* 5 ms) delivered on the surface of pyramids (spot H) in the VLM.

Fictive locomotion (FL) patterns were recorded from the left (l) and right (r) L2 VRs (flexor motor commands) and from l and r L5 VRs (extensor motor commands). Alternating discharges between homolateral L2 and L5 VRs and between homosegmental VRs are considered the distinct feature of FL (Kiehn 2006). FL was electrically evoked by trains of rectangular single pulses applied to DRrL6-S1 (160 single pulses at 2 Hz, pulse duration* =* 0.1 ms, intensity = 15–37.5 µA or 1.5–3.5 Thr) or to the pyramid in the VLM (trains of single pulses at 1–2 Hz, pulse duration* =* 1–5 ms, intensity = 0.5–4.5 mA).

### Serial Transection Experiments

In a subgroup of experiments, suprapontine structures were ablated from the whole CNS preparation by two serial horizontal transections. Firstly, precollicular decerebration was performed by surgically cutting the brain rostral to the fifth cranial nerves at the level of superior cerebellar arteries and caudal edge of inferior colliculi (Voituron et al. 2005). One hour later, a second transection was carried out at the level of the ninth cranial nerves to separate the pons and medulla. Before each cut, suction electrodes on cervical VRs were released to avoid any nerve damage and a new suction was adopted after cutting. In experiments with DR train stimulation, a further midthoracic transection (at the level of thoracic 4/5) was adopted to compare, in the same animal, FL in the intact CNS vs. the isolated spinal cord. Since in these experiments midthoracic transection did not affect the stability of lumbar VR signals, suctions were not released, thus allowing a direct comparison between the amplitude of signals before and after spinal transection.

### Tissue Oxygen Assessment

The whole-CNS preparation was warmed to 25–27 °C and allowed to stabilize for 30 min prior to PO_2_ measurements. Continuous sampling in our recording chamber was performed from 80 min until 4 hours from the induction of anesthesia, before any surgical procedures (total duration 160 min). PO_2_ levels showed that the solution at the surface of the bath was slightly more oxygenated than the one at the bottom, with PO_2_ levels of 559.42 ± 2.34 Torr at 300 µm under the surface of the oxygenated perfusing fluid, and 403.50 ± 6.89 Torr at 300 µm above the chamber floor (chamber depth = 4000 µm). Conversely, no differences were observed in PO_2_ along the length of the recording chamber.

Measurement of PO_2_ in both VLM and motor cortex was performed using a fiber-optic microsensor with a tip diameter of 50 μm (Optode, OxyMicro System, World Precision Instruments, FL, USA) implanted at a depth of 100 µm. The optode microsensor was mounted on a calibrated micromanipulator to enable fine control on the vertical plane. For VLM PO_2_ measurements, the tip of the microsensor was horizontally aligned with the pair of XII cranial nerves and placed equidistant from the lateral emergence of the XII nerve, and the midline. For cortical oxygen assessment, the tip of the microsensor was placed horizontally, in the middle of the longitudinal fissure, 2 mm lateral from the midline. PO_2_ measurements were taken every 1 s and were acquired directly by OxyMicro v7.0.0 software (OxyMicro System, World Precision Instruments, FL, USA). All PO_2_ measurements were instantly corrected for temperature, which was on average 25.59 ± 0.28 °C. Overall tissue oxygenation was calculated for each preparation as the average of all 5 min bins that were continuously sampled during the entire experiment (160 min). To assess any decay in tissue oxygenation during the entire experiment (160 min), we compared, for each preparation, the PO_2_ at the beginning (80 min; t_80_) and at the end of the continuous oxygen assessment (240 min; t_240_).

### Slice Immunostaining and Cell Counting

At the end of electrophysiological experiments, CNS preparations were fixed with 4% paraformaldehyde at 4 °C for overnight incubation. Tissue was then cryopreserved in 30% sucrose in water and stored at 4 °C for immunostaining following our standard procedure. Briefly, CNS preparations were cut into 30-µm-thick axial sections using a sliding cryostat microtome and incubated with a blocking solution containing: 5% fetal calf serum, 5% bovine serum albumin, and 0.3% Triton X-100 in PBS, for one hour at room temperature. Then, slices were incubated overnight at 4 °C with the antibodies: NeuN (1:200) and β-III tubulin (1:2000) for neurons, and glial fibrillary acidic protein (GFAP; 1: 500) for astrocytes (Taccola et al. 2010; Cifra et al. 2012; Deumens et al. 2013). Primary antibodies were visualized using the corresponding secondary fluorescent antibody (Alexa Fluor 488 or 544 at 1:500 dilutions; Invitrogen).

To visualize cell nuclei, slices were incubated for 30 min in 1 μg/ml solution of 4′, 6-diamidino-2-phenylindole (DAPI) and mounted using the Vectastain medium (Vector Laboratories, Burlingame, CA). After incubation with the secondary antibody, slides were finally visualized with a TCS SP2 Leica confocal microscope (Leica Microsystems Srl, Italy), epifluorescence microscopy (Zeiss Axioskop2, Carl Zeiss MicroImaging, Thornwood, NY) or Nis-Eclipse microscope (NIKON, Amsterdam, Netherlands) with 10 × and 20 × magnifications. NeuN, β-III tubulin, and GFAP-positive cell density were quantified in a region of interest (RoI) of 730 × 730 μm^2^ at 750 µm from the surface of the ventrolateral prefrontal cortex, using Image J software (http://imagej.nih.gov) on images at 20 × magnification. Note that the maximal thickness of the P0-2 brain was less than 4 mm.

When biomarker staining was diffuse, like with β-III tubulin and GFAP, signals were collected as mean fluorescence intensity, expressed in arbitrary units (AU) determined with densitometry analysis, using ImageJ software, in fields of 730 × 730 μm^2^ area.

### Data Analysis

To remove electrical interference, original traces were notched at 50 Hz through Clampfit 11.2 software (Molecular Devices Corporation, PA, USA). All spontaneous rhythmic motor discharges displaying large-amplitude depolarizations synchronous among bilateral VRs and appearing at regular intervals are ascribed to respiratory bursts. A burst is defined as a period of sustained membrane depolarization that originates with a rapid onset from the baseline and remains above a preset threshold (usually five times the standard deviation of baseline noise; Bracci et al. 1996). The time during which the membrane potential remains above the preset threshold is defined as burst duration (Bracci et al. 1996). Rhythmic discharges were also characterized based on their period, defined as the time between peaks of two consecutive cycles (Taccola and Nistri 2006; Dose et al. 2016). The ratio between standard deviation and mean value provides the coefficient of variation (CV), which is an index of consistency of responses (Taccola et al. 2020).

Mean electrically evoked reflex responses were obtained by averaging at least 15 traces not corrupted by any spontaneous activity.

Conduction velocity was calculated by dividing the time to peak of each response by the distance between stimulating and recording sites, as measured by a microcalibrated dial caliper (sensitivity = 20 µm).

Phase coupling between pairs of VRs was ascertained by the correlation coefficient function (CCF) using Clampfit 11.2 software. A positive CCF value ≥ 0.5 states that rhythmic signals from two VRs are synchronous, while CCF ≤ -0.5 accounts for alternating patterns (Taccola and Nistri 2005; Dose et al. 2016).

### Statistical Analysis

Statistical analysis was performed with GraphPad InStat 3.10 (Inc., San Diego, California, USA).

All data in boxplots show sample median (horizontal segment), 75th and 25th percentiles (top and bottom edges of box) and 1.5 times the interquartile range (whiskers). The number of animals is indicated as n in the Results, and data are reported as mean ± SD values. Before assessing statistical differences among groups, a normality test was performed to select the use of either parametric or nonparametric tests.

Accordingly, parametric data were analyzed with paired or unpaired t test, one-way analysis of variance (ANOVA) and repeated measures ANOVA, whereas Mann–Whitney test, Kruskal–Wallis test, and Wilcoxon matched-pairs signed-rank test were used for nonparametric data.

Multiple comparisons ANOVA was followed by Tukey–Kramer multiple comparisons test, Fisher’s LSD or Dunn’s method. Differences were considered statistically significant when *P ≤* 0.05.

## Results

### The Entire CNS In Vitro Displays a Spontaneous and Stable Fictive Respiratory Rhythm

**Fig. 2:**
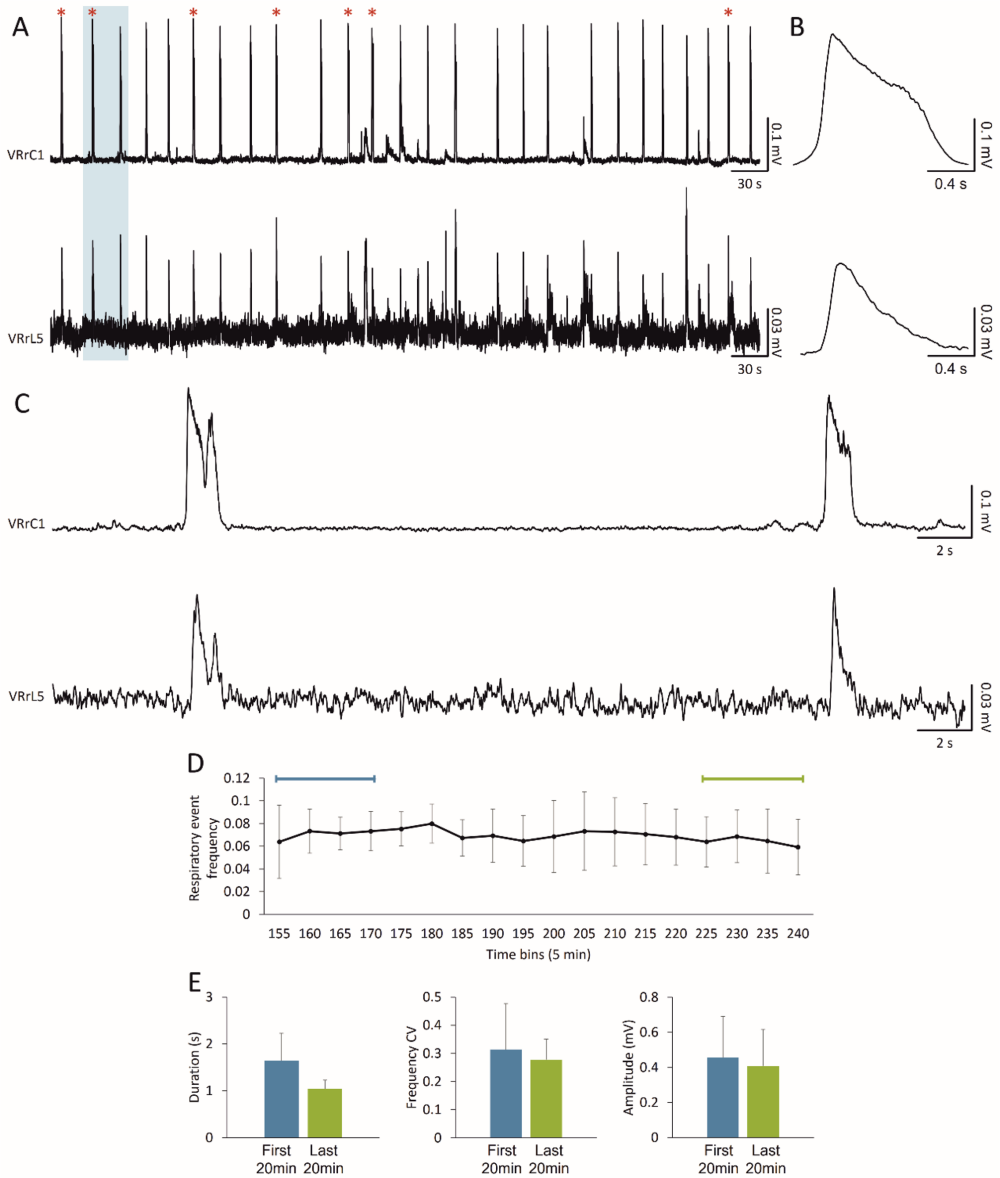
In vitro preparation of the entire CNS expresses 4 hours of stable fictive respiration. **A** Spontaneous and synchronous respiratory bursts are recorded for up to 4 hours from both cervical and lumbar VRs. The occurrence of sporadic double bursts is tagged by red stars. **B** Average bursts summarize shape and duration of cervical and lumbar respiratory events. **C** Magnification of traces in the shaded rectangle in A. A double and a single peak burst follow one another on both cervical and lumbar VRs. **D** The average time course shows the frequency of fictive respiration for the entire length of the experiment for 5 min bins (*n =* 5). The time indicated lapses from the induction of anesthesia. **E** Respiratory events in the first and last 20 min of the time course in (**D**) remain unchanged as for duration, regularity of rhythm expressed as frequency CV, and amplitude

The expression of spontaneous respiratory motor patterns is a sign of the functionality of neuronal networks in the brainstem–spinal cord in vitro (Smith et al. 1990). Likewise, in a sample preparation of the entire CNS isolated from newborn rats, we recorded respiratory-related rhythmic discharges at a frequency of 0.04 Hz, which appeared synchronous (CCF = 0.84) among cervical and lumbar ventral roots (Fig. 2A). The average bursts are reported in Fig. 2B, showing a duration of 1.47 s for VRrC1 and 1.09 for VRrL5 and a peak amplitude equal to 0.25 mV and 0.06 mV, respectively. Interestingly, double bursts only seldom appeared, as visualized in the magnification of Fig. 2C where double- (red star) and single-peaked respiratory events follow in a row.

Pooled data from twelve experiments indicate that, in the first 30 min of continuous recordings, the spontaneous respiratory rhythm has a frequency of 0.06 ± 0.03 Hz, with single bursts that last on average 1.80 ± 0.52 s and peak amplitude of 0.24 ± 0.13 mV. In six of those preparations, fictive respiratory events seldom appeared double-peaked (on average, 8.17 ± 2.48 double-peaked burst out of 102.17 ± 63.02 total respiratory events) and eventually turned into single bursts in the following 30 min. However, the sporadic occurrence of double bursts did not affect mean rhythm frequency (*P =* 0.448; unpaired t test), burst duration (*P =* 0.449; unpaired t test), nor peak amplitude (*P =* 0.240; Mann–Whitney test).

To prove the stability of the fictive respiratory rhythm derived from the entire CNS preparation in vitro, long recordings were continuously performed for at least 4 h right after tissue isolation. Figure 2D traces the time course of the mean rhythm frequency in 5-min bins for five experiments. Respiratory events recorded in the first 20 min were similar to the ones recorded at the end of the time course as for frequency (*P =* 0.536; paired t test), duration (*P =* 0.050; paired t test), frequency CV (*P =* 0.680; paired *t* test) and amplitude (*P =* 0.386; paired t test; Fig. 2E, Table 1).

**Table 1:** Features of fictive respiratory events at the onset and the end of electrophysiological experiments

	First 20 min	Last 20 min
Frequency (Hz)	0.07 ± 0.03	0.07 ± 0.02
Duration (s)	1.65 ± 0.59	1.04 ± 0.19
Frequency CV	0.31 ± 0.16	0.28 ± 0.07
Amplitude (mV)	0.46 ± 0.24	0.41 ± 0.21

The stable fictive respiratory rhythm recorded for more than 4 hours demonstrates that the entire CNS in vitro is a sound preparation for studying the respiratory network in a more intact experimental setting.

### Punctiform Electrical Stimulation of the Ventral Surface of the Brainstem and Pons Elicits Distinct Motor Responses Along Lumbar Segments

To assess descending input conduction along spinal segments in the CNS preparation, motor-evoked potentials were induced by serial pulses of electrical stimulation on different sites of the ventral brainstem. On each preparation, electrical pulses were delivered to ten loci on each side of the brainstem, ranging from higher pontine structures to the upper cervical cord (locations are detailed in the Methods section).

**Fig. 3:**
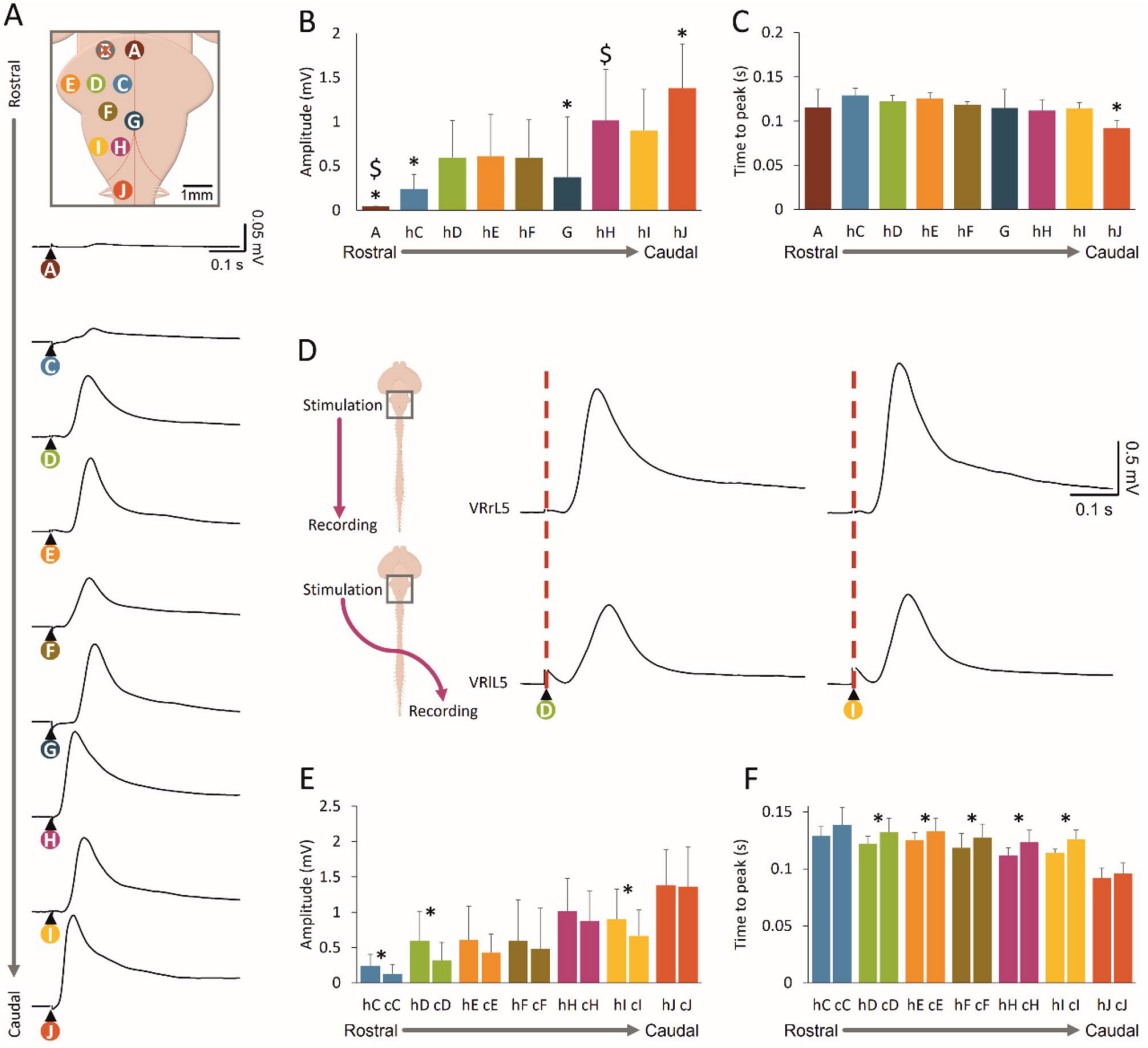
Distinct motor responses from lumbar VRs are evoked by punctiform electrical stimulation of the ventral surface of the brainstem and pons. **A** Different sites of pons and medulla were serially stimulated by a custom-made punctiform electrode (intensity = 2 mA, duration* =* 1 ms) and mean motor potentials were recorded from VRrL5. Each site of stimulation is indicated with a different color and letter in the schematic cartoon, which is calibrated to the real sample dimensions. Please note that only one side of the cartoon is labeled. Serial stimulation of distinct sites evokes motor responses of different amplitude (**B**, *n =* 5–8; *P < 0.001) and time to peak (**C**, *n =* 5–8; *P < 0.001). **D** Motor responses are elicited from r-l VRL5 by serially stimulating “D” (left panel) or “I” (right panel) spots on the right medulla (same experiment as in A). For each pulse, homolateral (upper) and contralateral (bottom) responses are simultaneously acquired. Average traces come from 15 superimposed sweeps. **E** Histogram reports the peak amplitude of homolateral and contralateral responses by serially stimulating different sites. Homolateral responses evoked by stimulation of “C” (*n =* 6; *P = 0.006), “D” (*n =* 8; *P = 0.010), and “I” (*n =* 7; *P = 0.023) are significantly higher than contralateral ones. (F) Histogram summarizes the time to peak of homolateral and contralateral responses when serially stimulating different sites. Homolateral responses to pulses applied to “D” (*n =* 8; *P = 0.043), “E” (*n =* 8; *P = 0.031), “F” (*n =* 7; *P = 0.020), “H” (*n =* 8; *P < 0.001), and “I” (*n =* 7; *P = 0.018) are significantly faster in comparison with contralateral

The cartoon in Fig. 3A identifies the stimulating sites using different letters and a color code. The responses displayed below were serially evoked, in the same preparation, by electrically stimulating distinct sites and were recorded from the homolateral VRrL5. In a sample experiment, the stimulating electrode was moved in a rostral–caudal direction, generating larger and more delayed responses (Fig. 3A). Medial rostral pons (indicated by the letter “A” in Fig. 3A) was the highest site of stimulation that still generated appreciable evoked potentials from lumbar motor pools (L5), with a peak of 0.04 mV and a time to peak of 0.14 s. The largest (Fig. 3B) and earliest (Fig. 3C) motor-evoked response, with a peak of 1.84 mV and a time to peak of 0.08 s, was obtained by pulses supplied to the ventrolateral surface of the first cervical spinal segment (named as J). On the other hand, the most efficient brainstem stimulation corresponded to the supply of pulses to the pyramid (as indicated by the letter “H”) with responses of 1.68 mV amplitude and 0.09 s time to peak. Contrariwise, in 17 preparations, spanning from 0- to 3-day-old newborns (5 preparations at P0; 4 at P1; 4 at P2 and 4 at P3) stimulation of lateral rostral pons and higher brain structures (dorsal cerebellum, ventral and dorsal mesencephalon, and cortex) always failed to elicit any reflexes from VRs (data not shown).

Average data describe responses evoked by serial multisite electrical stimulation in terms of amplitude (Fig. 3B) and time to peak (Fig. 3C) and report significantly larger responses from cervical spinal stimulation compared to rostral pulses (Fig. 3B; “A”, “C”, and “G”; *P <* 0.001; Kruskal–Wallis test, *n =* 5–8). Moreover, compared to all homolateral responses, the one originating from stimulation of the ″J″ site appears sooner (Fig. 3C; *P <* 0.0001; ANOVA test followed by Tukey–Kramer multiple comparisons test, *n =* 5–8).

The most rostral impulses that generated VRPs in three out of six experiments were delivered to the medial rostral pons (as indicated by the letter “A”), although responses were significantly lower compared to the ones elicited by pyramid stimulation (*P <* 0.001; Kruskal–Wallis test, *n =* 5–8).

To explore whether homolateral and contralateral stimulations evoked different spinal lumbar responses, simultaneous recordings were acquired from the right and left VRL5 when electrical pulses were serially applied to multiple sites of the brainstem–upper cervical cord. Figure 3D shows simultaneous recordings from bilateral VRL5 during electrical stimulation of the rostro-medial pons (“D”) and VLM (“I”). On both sites, responses were higher and appeared earlier for homolateral (peak_D_ = 1.20 mV, time to peak_D_ = 0.11 s; peak_I_ = 1.45 mV, time to peak_I_ = 0.11 s) vs. contralateral stimulation (peak_D_ = 0.77 mV, time to peak_D_ = 0.13 s; peak_I_ = 0.88 mV, time to peak_I_ = 0.11 s). Most stimulating configurations generated homolateral (h) and contralateral (c) evoked responses of unchanged amplitude, excluding the pons (“C” and “D”) and VLM (“I”; Fig. 3E; *n =* 5–8). As for latency of evoked responses expressed as time to peak, contralateral stimulation elicited delayed VRPs in all cases, except for the site indicated by the letter “C,” which is very close to the brainstem midline (Fig. 3F; *n =* 5–8). Average values are reported in Table 2.

**Table 2:** Amplitude of homolateral and contralateral VR potentials elicited by punctiform electrical stimulation of different spots on the ventral surface of the brainstem

Location	A (*n =* 3)	C (*n =* 6)	D (*n =* 8)	E (*n =* 8)	F (*n =* 7)	G (*n =* 6)	H (*n =* 8)	I (*n =* 7)	J (*n =* 8)
Homolateral peak amplitude (mV)	0.04 ± 0.00	0.24 ± 0.16	0.6 ± 0.48	0.61 ± 0.48	0.6 ± 0.43	0.37 ± 0.68	1.02 ± 0.58	0.9 ± 0.46	1.38 ± 0.5
Homolateral time to peak (m)	0.12 ± 0.02	0.13 ± 0.01	0.12 ± 0.01	0.13 ± 0.01	0.12 ± 0.00	0.12 ± 0.02	0.11 ± 0.01	0.11 ± 0.01	0.09 ± 0.01
Contralateral peak amplitude (mV)		0.13 ± 0.13	0.32 ± 0.25	0.43 ± 0.26	0.48 ± 0.37		0.88 ± 0.58	0.67 ± 0.42	1.36 ± 0.57
Contralateral time to peak (m)		0.14 ± 0.02	0.13 ± 0.01	0.13 ± 0.01	0.13 ± 0.01		0.12 ± 0.01	0.13 ± 0.01	0.1 ± 0.01
P value of homolateral and contralateral amplitude comparison		0.006 (paired t test)	0.010 (paired t test)	0.110 (Wilcoxon matched-pairs signed-ranks test)	0.097 (paired t test)		0.210 (paired t test)	0.023 (paired t test)	0.894 (paired t test)
P value of homolateral and contralateral time to peak comparison		0.143 (paired t test)	0.043 (paired t test)	0.031 (Wilcoxon matched-pairs signed-ranks test)	0.020 (paired t test)		< 0.001 (paired t test)	0.018 (paired t test)	0.123 (paired t test)

In summary, descending input recorded caudally from bilateral VRs of the lower lumbar cord shows the recruitment of specific functional pathways, having distinct latencies and motoneuronal recruitment mirroring the rostro-caudal supply of brainstem pulses.

### Descending Input Elicited by Brainstem Stimulation Travels Along the Cord with Different Conduction Velocity

**Fig. 4:**
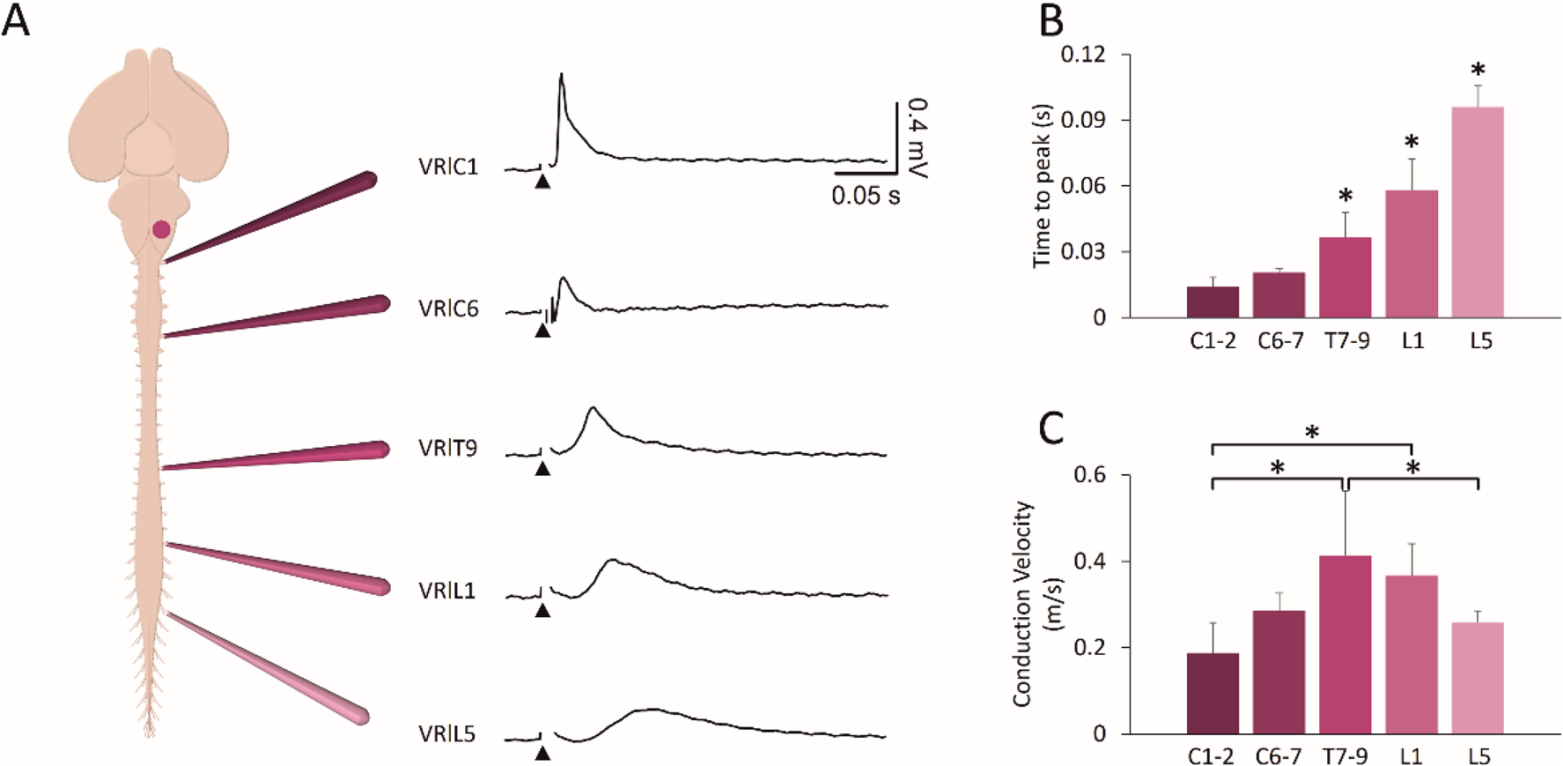
Electrical stimulation of the brainstem reveals different conduction velocities along the cord. **A** Single electrical pulses (intensity = 2 mA, duration* =* 1 ms) applied to the left pyramids of the brainstem (spot H) evoke simultaneous responses from cervical (C1, C6), thoracic (T9), and lumbar (L1, L5) VR on the left side of the cord. Average traces arise from 15 superimposed sweeps. **B** Histogram of the time to peak of responses shows that the slowest response comes from the most caudal segments (*n =* 5; *P < 0.001). **C** Histogram for conduction velocity of pulses follow a distinct trend (*P = 0.001)

To explore the organization of descending spinal pathways recruited by brainstem stimulation, electrical pulses were delivered to the pyramid (H spot) and responses taken from homolateral VRs at several spinal levels were used to calculate conduction velocity. In Fig. 4A, evoked motor potentials were acquired from five spinal segments (from C1 to L5) in correspondence to the stimulation of the left medulla. Responses became lower and slower when recorded from more caudal spinal segments. While the upper cervical peak appeared sooner (C1; 0.02 s) and was more similar to lower cervical regions (C6; 0.02 s), the thoracic VR response was delayed (T9; 0.04 s) and even more delayed were responses derived from upper (L1; 0.05 s) and lower (L5; 0.09 s) lumbar segments.

Data collected from 5 experiments demonstrate that potentials recorded from thoracolumbar segments appear later than cervical responses (in Fig. 4B; *P <* 0.001; ANOVA). Pulse conduction velocity based on the actual distance between each pair of stimulating and recording sites revealed that the input descending to thoracic segments is the fastest, and that the input to lumbar segments is faster than the cervical one (Fig. 4C; *P =* 0.001; ANOVA; Table 3).

**Table 3:** Time to peak and conduction velocity of spinal motor responses evoked by electrical stimulation of left ventrolateral medulla

	C1/C2	C6/C7	T7/8/9	L1	L5
Time to peak (s)	0.01 ± 0.00	0.02 ± 0.00	0.04 ± 0.012	0.06 ± 0.01	0.1 ± 0.01
Conduction velocity (m/s)	0.19 ± 0.07	0.29 ± 0.04	0.4 ± 0.15	0.37 ± 0.07	0.26 ± 0.03

The different conduction velocity of descending input elicited by brainstem stimulation suggests that brainstem stimulation enrolls a propriospinal network with distinct synaptic relays.

### Electrical Stimulation of Caudal Dorsal Roots Evokes Ascending Input Along the Cord

**Fig. 5:**
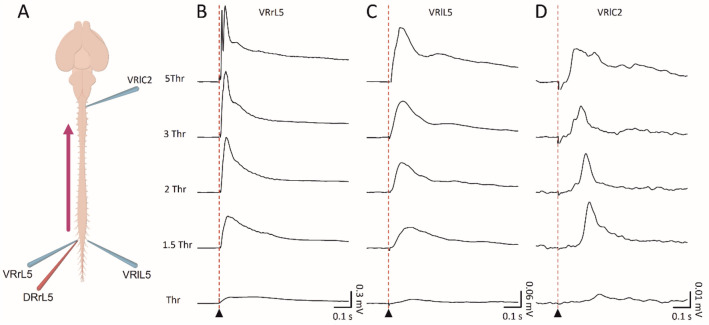
Input/output caudal stimulation elicits evoked motor responses along the cord. Electrical pulses (intensity = 0.02–0.1 ms, duration* =* 0.1 ms) delivered to lumbar afferents of the L5 segment evoke responses from the homologous VRL5 (**B**), contralateral VRL5 (**C**) and contralateral higher cervical VRC2 (**D**). By increasing the strength of stimulation, responses appear sooner and higher. Average traces have been pooled from 15 superimposed sweeps

After describing the conduction of descending input evoked by brainstem stimulation through caudal segments of the whole CNS in vitro, we sought to properly characterize the transit of ascending input. For this purpose, electrical pulses were delivered to caudal afferents while monitoring motor responses from rostral segments. In the sample experiment schematized by the cartoon in Fig. 5A, brief pulses (duration* =* 0.1 ms) were serially applied (0.03 Hz) to a lower lumbar (L5) DR, simultaneously recording responses from homologous and contralateral VRs and from a rostral cervical segment (C2). Input/output stimulation was supplied at augmenting intensities, expressed as multiples of the Thr calculated from the homologous lumbar VR (1 x, 1.5 x, 2 x, 3 x, 5 × Thr), evoking increasingly larger and faster potentials from all spinal VRs. At all strengths of stimulation, motor-evoked responses were higher from the homologous VR (Fig. 5B) than from the contralateral L5 (Fig. 5C, note the lower scale on the y-axis). Motor-evoked responses were also induced from upper cervical segments, albeit with lower and delayed potentials (Fig. 5D).

Input/output average values for each VR are reported in Table 4 for amplitude and time to peak (*n =* 7). Increases in stimulation strengths correspond to higher average peaks of lumbar DRVRPs, while cervical responses remain less affected. The average time to peak of all reflexes was slightly reduced at the first step of increasing stimulation (1 × Thr Vs 1.5 × Thr), while it remained unchanged for stronger pulses. Conduction of ascending input along the cord is maintained in the isolated CNS preparation, with rostral VRs far from the stimulating segment showing smaller responses with a later onset. However, no responses from the surface of dorsal motor cortices were acquired in correspondence to dorsal stimulation, even at higher strengths and durations of pulses (data not shown), reminiscent of the above-mentioned inability of the motor cortex to elicit spinal responses through electrical stimulation.

**Table 4:** Input–output experiments for electrical stimulation of DRrL5

Root		Thr	1.5 Thr	2 Thr	3 Thr	5 Thr
VRrL5	Amplitude (mV)	0.53 ± 0.5	0.87 ± 0.61	0.80 ± 0.47	0.84 ± 0.41	0.89 ± 0.42
	Time to peak (s)	0.05 ± 0.02	0.03 ± 0.02	0.03 ± 0.02	0.03 ± 0.02	0.03 ± 0.02
VRlL5	Amplitude (mV)	0.23 ± 0.29	0.45 ± 0.34	0.58 ± 0.48	0.67 ± 0.56	0.7 ± 0.59
	Time to peak (s)	0.08 ± 0.03	0.07 ± 0.02	0.07 ± 0.02	0.07 ± 0.02	0.07 ± 0.02
VRlC2	Amplitude (mV)	0.18 ± 0.15	0.16 ± 0.1	0.18 ± 0.12	0.20 ± 0.15	0.20 ± 0.13
	Time to peak (s)	0.12 ± 0.03	0.12 ± 0.02	0.12 ± 0.02	0.12 ± 0.02	0.12 ± 0.02

### Pulse Oximetry Optical Assessments Show Preserved and Stable Oxygen Levels in the Cortex

**Fig. 6:**
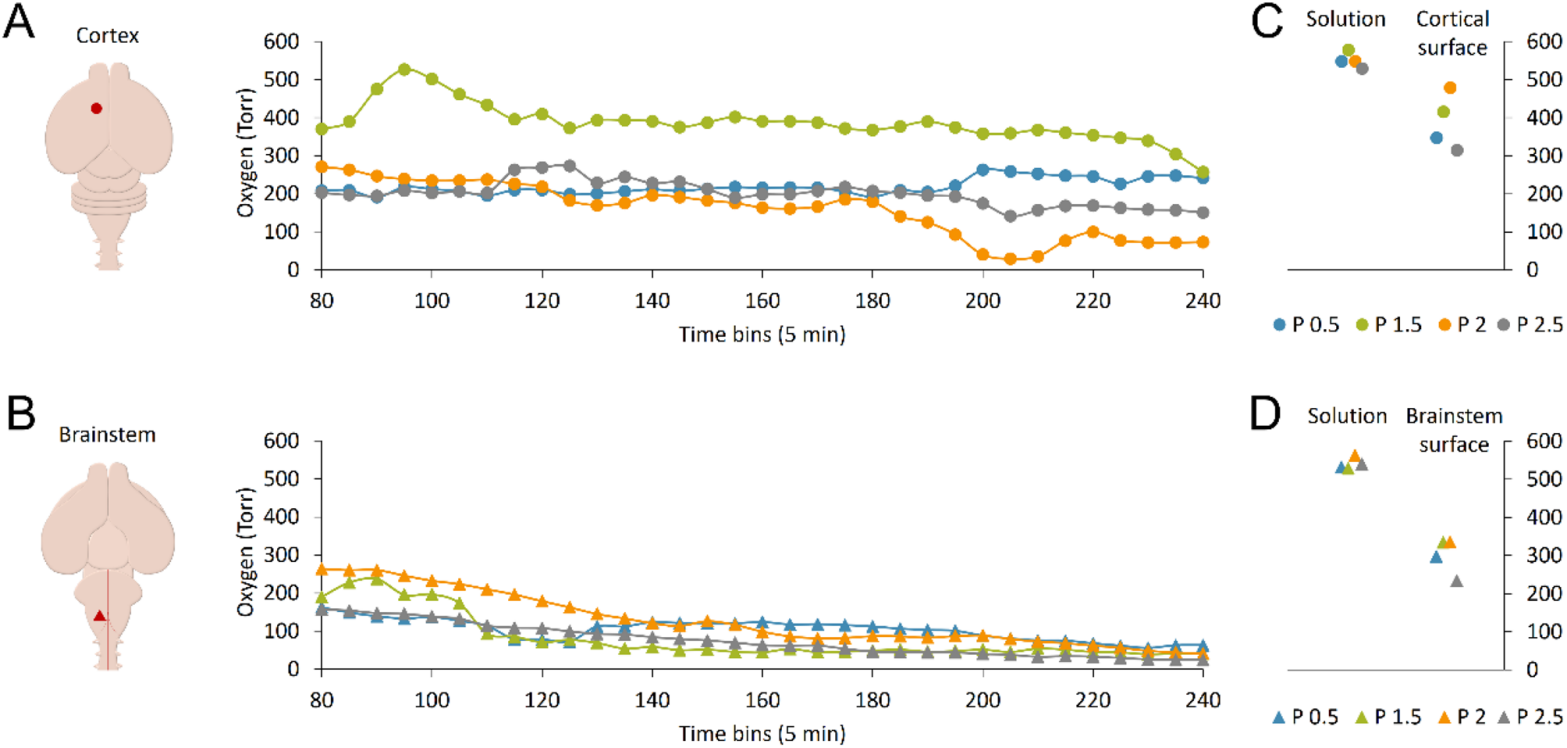
Tissue oxygen levels in the cortex are higher than in the brainstem at all ages explored. In eight different preparations, PO_2_ is continuously monitored at a 100 µm depth on the motor cortex (**A**) and brainstem (**B**). Left cartoons show the sites of the probe implant in the cortex (**A**) and brainstem (**B**). Time courses indicate the average PO_2_ levels in 5 min bins for the entire duration of experiments, from 80 min to 4 h from anesthesia (circles in A for cortex, and triangles in B for brainstem). Each preparation is traced with different hues adopting the color code reported in (**C, D**). Right plots show the average PO_2_ in the solution and on the surface of the cortex (**C**) and brainstem (**D**) for all preparations tested

Electrophysiological data exposed so far show a stable spontaneous respiratory activity from higher cervical VRs and an optimal pulse conduction along the brainstem–spinal cord axis. However, no motor responses were elicited by electrically stimulating the surface of the cortex or midbrain, as well as no surface potentials were recorded from the same sites following lumbosacral DR stimulation. To verify whether this lack of responses derives from hypoxic conditions of brain structures due to the time-consuming surgical procedures and the long maintenance in vitro, in a subset of eight preparations isolated from rats of 0.5, 1.5, 2, and 2.5 postnatal days, we continuously measured PO_2_ through an Optode microsensor implanted 100 µm deep in either the motor cortex or the brainstem. Time courses show the dynamics of PO_2_ during the entire experiment for each preparation, recorded either from the motor cortex (Fig. 6A, *n* = 4) or from the brainstem (Fig. 6B, *n* = 4). For all experiments, PO_2_ was on average 545.83 ± 17.34 Torr (*n =* 8) in the bath solution, while it dropped to 388.98 ± 73.18 Torr for the cortex (Fig. 6C) and 300.03 ± 47.85 Torr for the brainstem (Fig. 6D) when measured on the tissue surface. Interestingly, 100 µm below the tissue surface, overall oxygenation of the cortex (240.55 ± 101.54 Torr, *n =* 4) was higher than the brainstem’s (94.58 ± 23.70 Torr, *n =* 4; *P =* 0.031, t test).

In addition, for all ages explored, a common decaying trend in tissue oxygenation of the brainstem occurred over time (t_80_ = 197.93 ± 55.06 Torr Vs t_240_ = 42.53 ± 15.97 Torr; *P =* 0.013, paired t test, *n =* 4), while cortical PO_2_ values remained stable throughout the entire experiment (t_80_ = 264.75 ± 88.17 Vs t_240_ = 180.66 ± 85.45; *P =* 0.184, paired t test, *n =* 4).

### Histological Analysis of Neurons and Glial Cells Demonstrates Well-Structured Tissue Preservation In Vitro for Over 4 Hours

**Fig. 7:**
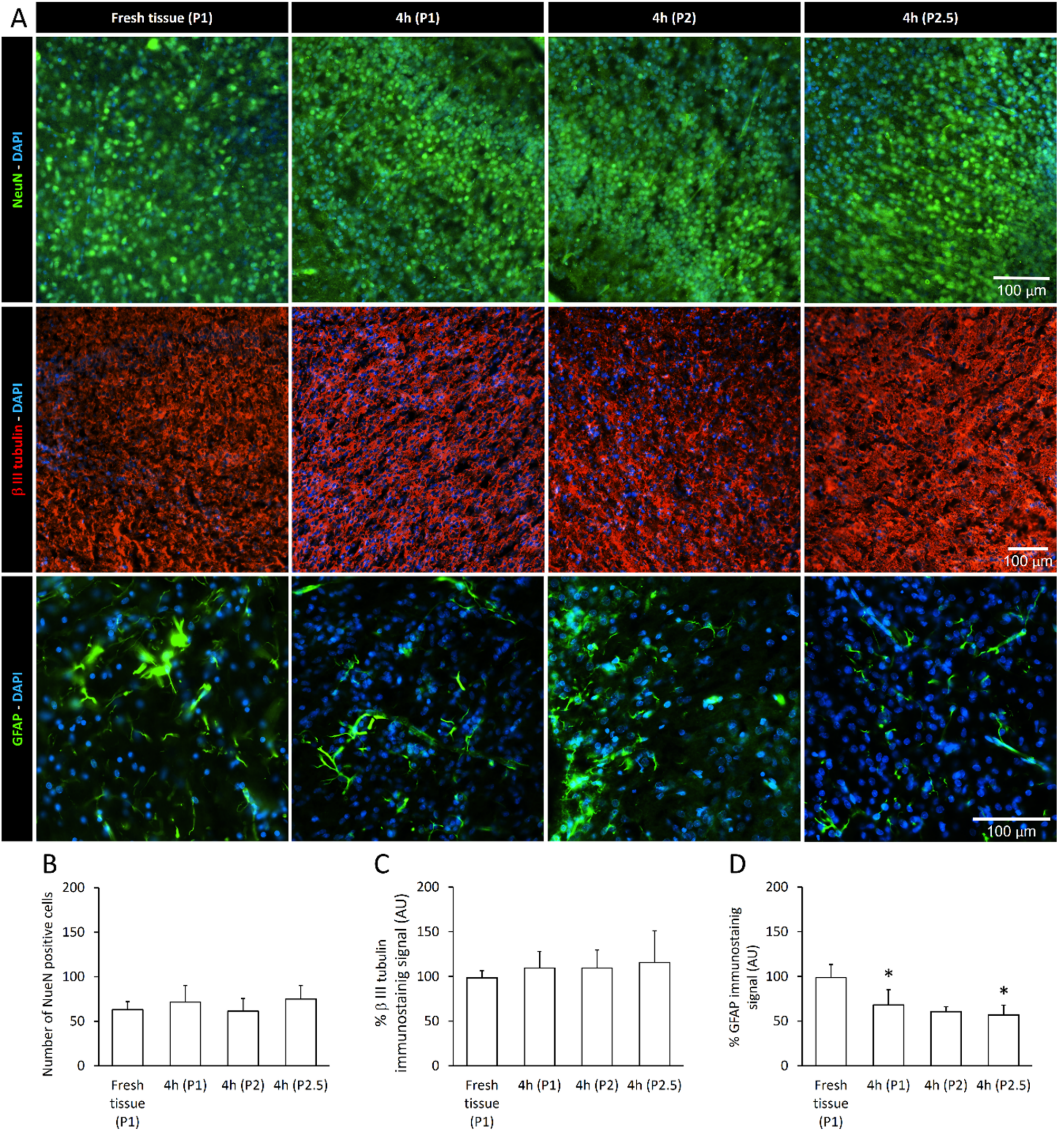
Neuronal and glial staining of internal brain structures under the surface of the ventrolateral prefrontal cortex from the CNS in vitro. **A** Examples of NeuN (upper panels), β-III tubulin (middle panels) or GFAP staining (lower panels) at 750 µm from the surface of the ventrolateral prefrontal cortex. **B** Histogram shows the mean number of NeuN positive cells in RoIs of 730 × 730μm^2^, showing no statistical difference among fresh tissue and the three preparations kept 4 h in vitro. **C–D** Histograms show mean β-III tubulin (**C**) and GFAP (**D**) in RoIs of 730 × 730μm^2^, expressed as fluorescence intensity in arbitrary units (AU, percentage change from fresh tissue). No significant differences in β-III tubulin signal are apparent among fresh tissue and the 3 preparations kept 4 h in vitro. GFAP fluorescence intensity quantification for astrocytes indicates a decrease in immunoreactivity in two preparations kept 4 h in vitro (D; *P = 0.0002)

In Fig. 7, the topographical distribution of neuronal and non-neuronal cells was analyzed in brain structures at 750 µm from the surface of the ventrolateral prefrontal cortex, from one fresh tissue right after isolation of the entire CNS (P1) and from three different CNS preparations (P1–P2.5) maintained in the warmed recording chamber (25–27 °C) for the entire duration of experiments (4 h).

The average total number of neuronal cells, as labeled by NeuN in an RoI of 730 × 730 µm^2^, is shown in Fig. 7A (green, first row) and quantified in B. The resulting mean density of NeuN positive cells, expressed as 10^–4^/μm^2^, was similar among fresh tissue (1.18 ± 0.04) and the three different CNS preparations (1.36 ± 0.12; 1.17 ± 0.07; 1.43 ± 0.05; *P >* 0.05, One-way ANOVA on ranks followed by all pairwise multiple comparisons with Dunn's method). Similarly, β-III tubulin immunoreactivity (red, Fig. 7A middle row, C) was similar among preparations.

To identify any stress of the tissue after 4 h in vitro, GFAP was targeted as a marker for the activation of astrocytes (Verkhratsky and Parpura 2016). The mean fluorescence intensity for GFAP immunoreactivity was analyzed in fresh tissue and three preparations. Our results did not show any increase in GFAP immunoreactivity, but in fact demonstrated a significant reduction in two out of three preparations kept 4 h in vitro (Fig. 7D, *P =* 0.0002, ANOVA followed by Kruskal–Wallis test versus fresh tissue; *n =* 3–11 slices).

These results demonstrated no cell death and absence of astrogliosis in the internal brain structures under the surface of the ventrolateral prefrontal cortex in the preparation of the entire CNS, despite the long maintenance in vitro. This model thus appears suitable for exploring the modulatory influences of brain centers on the brainstem and spinal networks.

### Suprapontine Structures Modulate Neuronal Networks for Respiration

**Fig. 8:**
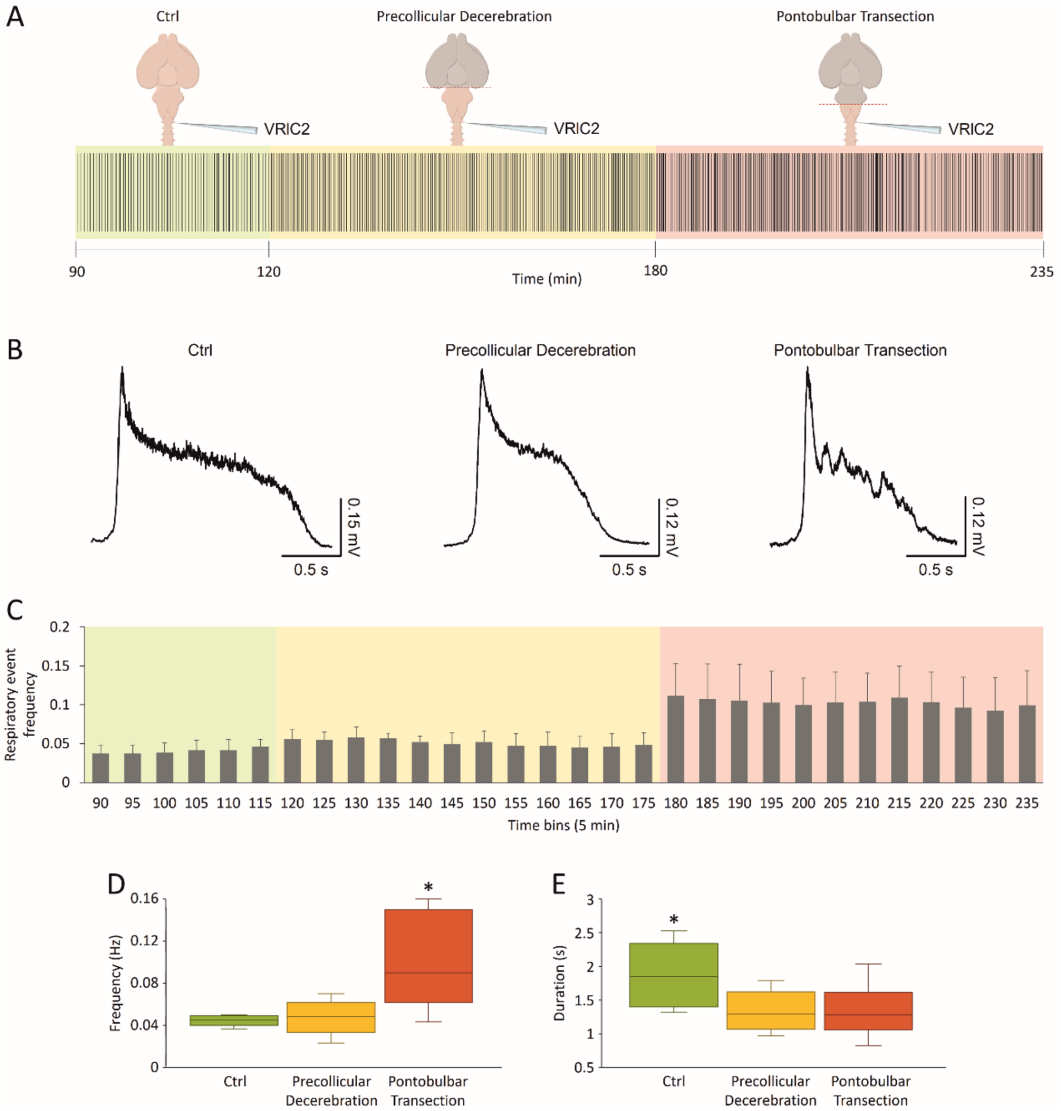
Fictive respiration is modulated by suprapontine and pontomedullary structures. (A) Raster plot of continuous fictive respiration recorded from VRlC2 for an entire experiment. Time is calibrated at the onset of anesthesia. Fictive respiration in control (green pale background) remains stable after a precollicular decerebration (yellow pale background) and eventually becomes faster after the following pontobulbar transection (red pale background). **B** Average bursts from the same experiment in (**A**) are reported for the last five minutes of recordings in each experimental slot. Single events become shorter after precollicular decerebration, without any changes after the following pontobulbar transection. **C** An average time course from 8 experiments traces rhythm frequency in 5 min bins for the entire duration of experiments. The last five minutes of each experimental phase in C is used for statistical comparison of rhythm frequency (**D**, *P = 0.002) and burst duration (**E**, *P = 0.016)

To better characterize the impact of higher rostral centers on the neuronal pathways involved in respiration, a spontaneous respiratory rhythm was derived initially from upper cervical segments in the whole CNS in vitro and then after precollicular decerebration followed by the ablation of pons (Fig. 8A). In a sample preparation, the dynamics of respiratory events were described throughout the entire experimental protocol (2.5 h; Fig. 8A, raster plot). The stable respiratory rhythm frequency (green field) was not affected by precollicular decerebration (yellow field), while it was speeded up after the following pontobulbar transection (red field). Average bursts were obtained by superimposing the events in the last 5 min of each phase of the experiment (Fig. 8B). Single respiratory bursts in the intact CNS (1.88 s; left) shortened after precollicular decerebration (1.24 s; middle) and remained short after pontobulbar transection (1.03 s; right).

The time course of the mean frequency from eight experiments is reported for 5 min bins in Fig. 8C. Frequency of the respiratory rhythm increased after pontobulbar transection (Fig. 8D; *P =* 0.002; repeated measures ANOVA followed by Tukey–Kramer multiple comparisons test), while burst duration was already reduced after precollicular decerebration (Fig. 8E; *P =* 0.016, repeated measures ANOVA followed by Tukey–Kramer multiple comparisons test).

Collectively, data indicate that suprapontine structures affect distinct features of the fictive respiratory rhythm, supporting the adoption of the whole CNS in vitro preparation to clarify the rostral modulation of brainstem networks.

### The Isolated CNS with Legs Attached Expresses a Stable Spontaneous Fictive Respiration Modulated by Suprapontine Structures

**Fig. 9:**
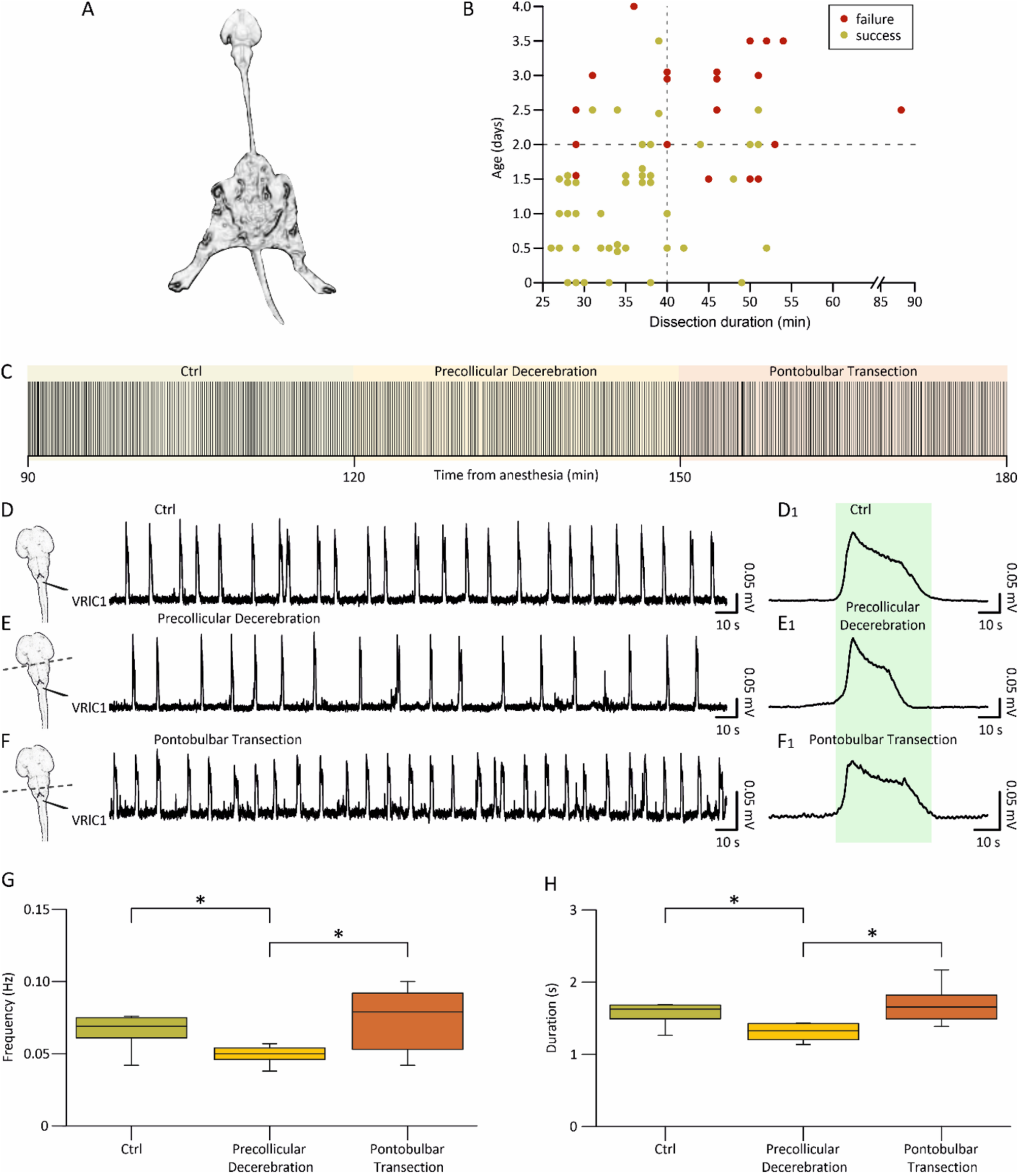
In vitro preparation of the entire CNS with hindlimbs attached expresses a stable fictive respiration that is affected by precollicular and pontomedullary serial transections. **A** Picture of the original in vitro preparation from a neonatal rat (P2), comprising the whole central nervous system with hindlimbs attached. Lower extremities and tail were left intact, along with ventral roots (VRs) and dorsal roots (DRs) below T13 segment. **B** Plot depicts each preparation as a single dot (66 preparations) describing age of the animal (Y-axis) and length of surgical dissection (X-axis). Dots are colored in green (success) or red (failure) based on the presence or absence of a stable respiratory rhythm after at least 4 h from the induction of anesthesia at the beginning of tissue isolation. Vertical dotted gray line at x = 40 min and horizontal dotted gray line at y = 2 days, define a bottom-left quadrant where the probability of having preparations with long-lasting breathing is highly consistent (44 successes out of 47 preparations). **C** Raster plot representing consecutive respiratory bursts continuously recorded from a cervical VR (lC1) in the intact CNS (Ctrl; 30 min; green shadow field) and after serial precollicular (30 min; yellow shadow field) and pontobulbar transections (30 min; red shadow field). Albeit the expected variability, rhythm frequency is reduced after decerebration, while it is increased by the subsequent pontomedullary transection. For the same experiments reported in (**C**), three trace segments from VRlC1 are taken at steady state in intact settings (**D**) and during the progressive reductions of the intact CNS preparation (**E**, **F**; see left cartoons). Burst frequency in the entire CNS (top trace) is slowed down by decerebration (middle trace) and then eventually speeded up after pontomedullary transection (bottom trace). (**D**_**1**_**, E**_**1**_**, F**_**1**_) Average bursts calculated by superimposing single events are reported on the right, demonstrating that decerebration reduces burst duration, which eventually recovers after further tissue ablation. Differences in burst duration are highlighted by the green shaded field corresponding to the burst duration calculated in control. Effect of rostral structures ablation on the frequency of respiration is summarized by whisker plots from pooled data in G (*n =* 9), showing significant changes in the pace of rhythm from the intact CNS (green box, ctrl, *n =* 9) following precollicular decerebration (yellow box, precollicular transection, *n =* 9) and then pontomedullary transection (orange box, pontobulbar decerebration, *n =* 6; *P = 0.036). **H** Whisker plots describing changes in burst duration from the intact CNS (green box, *n =* 11) following serial transections (yellow box, precollicular transection, *n =* 11; orange box, pontobulbar decerebration, *n =* 8; *P = 0.003). Note that burst amplitude has not been evaluated since nerves were released from electrodes before each surgical ablation to avoid root damage

Isolated preparations of brainstem and spinal cord with hindlimbs kept intact and connected to the spine have been used to explore both the functional coupling between networks for respiration and locomotion (Giraudin et al. 2012), as well as the modulatory influence on spinal circuits played by the afferent feedback elicited by passive exercise (Dingu et al. 2018). However, to explore whether suprapontine structures contribute to integrating the afferent input elicited by passive leg movement, the definition of a more intact in vitro preparation became compelling. To this purpose, we isolated the whole CNS keeping the entire hindlimbs connected to the spine (Fig. 9A). This semi-intact preparation expresses a stable spontaneous respiratory rhythm for over 4 h when surgical procedures for tissue isolation are fast (≤ 40 min) and performed on younger animals (≤ 2 days old), as summarized in the scatter plot of Fig. 9B from 66 preparations. A sample respiratory rhythm from a 2-day-old neonatal preparation (raster plot in Fig. 9C) was progressively slowed down by precollicular decerebration (Fig. 9C, yellow field) and then speeded up by the following pontobulbar transection (Fig. 9C, red field). Original traces at steady state illustrate the regular bursting at 0.08 Hz in the intact preparation (Fig. 9D), which slowed down to 0.06 Hz after precollicular transection (Fig. 9E) and then accelerated to 0.10 Hz following ablation of the pons (Fig. 9F). In the intact preparation, the average single burst lasted 1.63 s (Fig. 9D1) and was reduced to 1.43 s after ablation of suprapontine structures (Fig. 9E1), while pontobulbar transection broadened burst duration to 1.84 s (Fig. 9F1). Pooled data from many experiments confirm the significant reduction in the bursting frequency after precollicular decerebration (from 0.07 ± 0.02 Hz to 0.05 ± 0.02 Hz; *P =* 0.036; one-way ANOVA followed by all pairwise multiple comparisons with Fisher’s LSD method; *n =* 9) and its subsequent recovery after pontobulbar transection (0.07 ± 0.02 Hz; *P =* 0.036; one-way ANOVA followed by all pairwise multiple comparisons with Fisher’s LSD method; *n =* 6). Similarly, burst duration was reduced by decerebration (from 1.72 ± 0.46 s to 1.22 ± 0.43 s; *P =* 0.003; one-way ANOVA on ranks followed by all pairwise multiple comparisons with Dunn's method; *n =* 11) and then broadened again after the following pontobulbar transection (1.68 ± 0.25; *P =* 0.003; one-way ANOVA on ranks followed by all pairwise multiple comparisons with Dunn’s method; *n =* 8).

In summary, hindlimbs kept attached to the isolated CNS do not affect the expression of the spontaneous respiratory rhythm, nor its modulation provided by suprapontine structures. Thus, the whole CNS with legs attached represents an original setting to explore how respiration is tuned by afferent input reaching the brain from the periphery.

### Suprapontine Structures Modulate Local Lumbar Circuitry

We thus explored whether the presence of suprapontine structures in the CNS preparation affects spinal motor networks in the lumbar cord. To characterize the state of excitability of spinal motor networks, we used dorsally evoked potentials derived from VRs in response to single electrical pulses supplied to dorsal afferents (Lev-Tov and Pinco 1992). Interestingly, afferent dorsal pulses also recruit a specific dorsal network along the cord (Taccola and Nistri 2005) that is involved in the presynaptic inhibition of input coming from the periphery (Rudomin 2009).

**Fig. 10:**
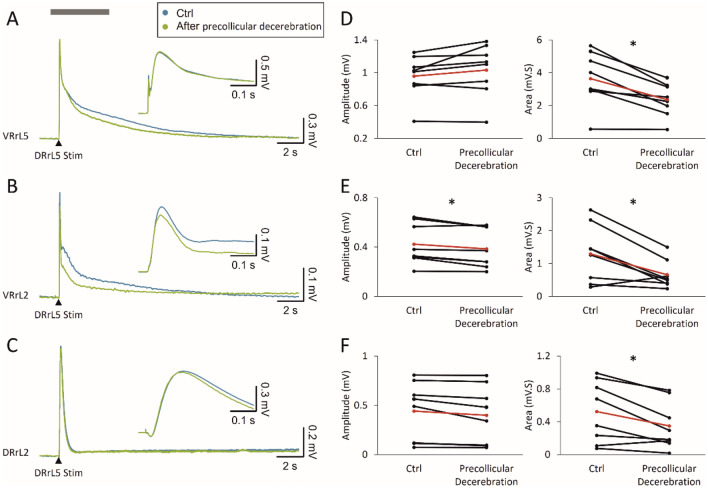
Local lumbar networks involved in reflex responses are modulated by suprapontine structures. Superimposed average traces show that electrical pulses applied to a DRlL5 (intensity = 45 µA, pulse duration* =* 0.1 ms) induce both VR and DR potentials from VRrL5 (**A**), VRrL2 (**B**) and DRrL2 (**C**). After acquiring evoked responses in control (blue traces), the same stimulating protocol is repeated after precollicular decerebration (green traces). Inserts show the magnified onset of the response to better appreciate any changes in peak amplitude after decerebration. **D–F** For each recording site, pairs of responses before and after decerebration are quantified for amplitude and area. Red dots correspond to the mean value in each graph (*n =* 8; **D**, *P = 0.003; **E**, amplitude *P = 0.016, area *P = 0.039; **F**, *P = 0.016)

As shown in the sample mean traces reported in Fig. 10A and B, electrical stimulation (6–60 µA 2–3.3 Thr, 100 µs) of a DRlL5 in the isolated CNS elicits an early sharp peak from VRs coming essentially from an oligosynaptic pathway in the local microcircuitry, and a following long potential corresponding to the activation of a larger number of interneurons. A sharper potential is induced also from DRlL2 (Fig. 10C), following antidromic conduction of the primary afferent depolarization elicited by DRlL5 stimulation. Sample average traces in Fig. 10A–C were acquired before and after a precollicular transection, indicating that decerebration causes a faster decay of all responses and a 23% reduction in the peak of responses from VRrL2 (Fig. 10B).

Reflexes were acquired from both the intact CNS preparation (blue lines) and after precollicular decerebration on the same sample (green lines). As summarized in the plots of Fig. 10D–F, decerebration does not affect peak amplitude of rhythms acquired from VRrL5 (*P =* 0.103, paired, *t* test; *n =* 8) or DRrL2 (*P =* 0.055, paired, *t* test; *n =* 8), while peak amplitude of the VRrL2 rhythm is reduced (*P =* 0.016 paired, t test; *n =* 8).

Similarly, the area of all responses is depressed after precollicular transection (Area_VRrL2_ *P =* 0.039, Wilcoxon matched-pairs signed-ranks test; Area_VRrL5_ *P* = 0.003, paired *t* test; Area_DRrL2_ *P =* 0.016 paired t test; *n =* 8).

These observations demonstrate that the lack of suprapontine structures deprives lumbar circuits of some modulatory influences, suggesting the adoption of the whole in vitro CNS preparation whenever interested in exploring supraspinal influences on spinal microcircuits.

### Fictive Locomotor Patterns are Induced by Trains of Electrical Pulses Applied to Both Caudal Afferents and Ventrolateral Medulla

**Fig. 11:**
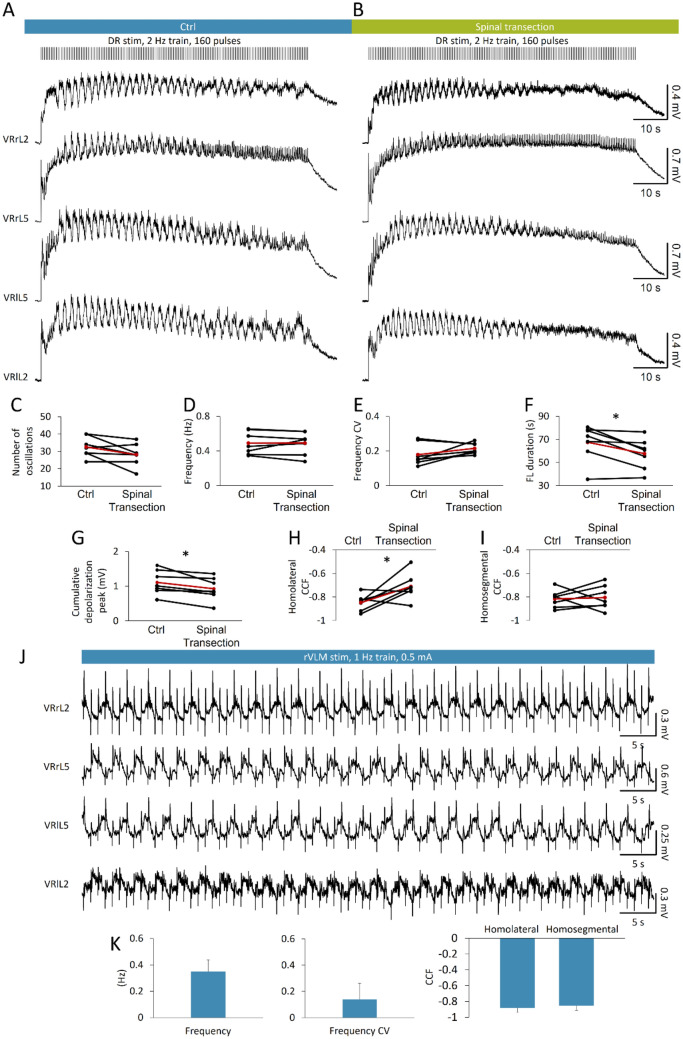
Fictive locomotor patterns are elicited by repetitive electrical stimulation applied either to a lumbar DR or to the ventrolateral medulla. An epoch of FL is induced by a train of pulses (160 stimuli, 2 Hz, intensity = 22.5 µA, pulse duration* =* 0.1 ms) applied to DRrL6 both in control (**A**) and after a midthoracic transection (**B**). VR oscillations appear double alternated between homolateral L2 and L5 segments, and between homosegmental left and right motor pools. Pooled data from seven experiments indicate that, after a midthoracic transection, there are significant differences in fictive locomotion (FL) duration (**F**, *P = 0.027), cumulative depolarization peak amplitude (**G**, *P = 0.023) and homolateral CCF (cross-correlation function; **H**, *P = 0.038), with an unchanged number of oscillations (**C**, *P = 0. 075), frequency (**D**, *P = 0.974), frequency CV (**E**, *P = 0.127) and homosegmental CCF (**I**, *P = 0.797). Red dots indicate the mean values in each graph. **J** Fictive locomotion is stably evoked by the continuous repetitive stimulation (1 Hz) of the right ventrolateral medulla (rVLM, “H” site; intensity = 0.5 mA, pulse duration* =* 1 ms). **K** Bars describe period, frequency CV homolateral and homosegmental CCFs of fictive locomotion oscillations as an average of four experiments

Fictive locomotion (FL; Kiehn 2006) consists in rhythmic electrical oscillations alternating at the segmental level between the two sides of the cord and, on the same side, among flexor and extensor motor pools. FL is a distinctive feature of the activation of the neuronal circuits for locomotion in the spinal cord preparation isolated from rostral thoracic segments to the cauda equina (Cazalets et al. 1992). To ascertain whether the recruitment of locomotor spinal networks in vitro is preserved even in the presence of suprapontine structures, we applied the canonical pattern of electrical stimulation (stereotyped trains of brief rectangular pulses at 2 Hz; Marchetti et al. 2001) to a caudal afferent of the entire isolated CNS. Sample traces in Fig. 11A were taken after about 1.5 hours from the beginning of the surgical procedures required for the isolation of the entire CNS, and show a cumulative depolarization evoked from four lumbar VRs when an 80 s train of rectangular pulses (duration* =* 0.1 ms) at 2 Hz (blue bar) was applied to DRrL6. At the top of the cumulative depolarization appeared an epoch of 34 rhythmic discharges in VRlL5, with a mean frequency of 0.45 Hz, that alternated between the flexor-related VRL1 and the extensor-related VRL5 on the right side of the cord, and between bilateral VRL5s. In the same preparation, after transecting the spinal cord at T3/T4 level (Fig. 11B, green bar), the same stimulating protocol elicited an episode of FL provided of 28 rhythmic oscillations with a mean frequency of 0.49 Hz in VRlL5. CCF analysis suggests a stronger phase coupling in the intact preparation (CCF_lL2-rL2_ = − 0.78, CCF_lL2-lL5_ = − 0.85) than after midthoracic transection (CCF_lL2-rL2_ = − 0.65, CCF_lL2-lL5_ = − 0.66).

In seven preparations, FL episodes were evoked by a train of 160 pulses at 2 Hz (intensity = 7.5–37.5 µA, 1.5–3.5 Thr; pulse duration* =* 0.1 ms) applied to lumbosacral afferents (DRrL6, DRrS1). Episodes of FL were equal before and after a midthoracic transection, as for mean number of locomotor-like oscillations (Fig. 11C; *P =* 0.075, paired t test), mean frequency of oscillations (Fig. 11D; frequency: *P =* 0.974, paired t test), mean variability of cycles (Fig. 11E; frequency CV: *P =* 0.127, paired t test), and left/right alternation (Fig. 11I; homosegmental CCF: *P =* 0.797, paired t test).

However, the total duration of FL episodes (67.50 ± 15.82 s vs. 57.61 ± 13.37 s; Fig. 11F; *P =* 0.027, paired *t* test), mean cumulative depolarization peak (Fig. 11G; *P =* 0.023, paired *t* test), and flexor/extensor alternation (Fig. 11H; *P =* 0.038, paired *t* test) were significantly reduced after disconnection from the brain. As opposed to transient epochs of FL patterns evoked in the isolated spinal cord, which spontaneously decayed despite the continuous presence of stimulation (Dose et al. 2014), more stable locomotor-like oscillations were observed in the brainstem–spinal cord preparation in response to a train of electrical pulses applied to the VLM (Zaporozhets et al. 2004). To verify whether electrical stimulation of the VLM induces stable FL patterns in the entire CNS preparation, a train (intensity = 0.5 mA, duration* =* 1 ms, frequency = 1 Hz) of pulses was continuously applied to the VLM for a total duration of 12 min. In response to stimulation, stable discharges appeared at 0.33 Hz, alternating between homolateral extensor and flexor output (CCF = -0.96) and homosegmental left and right ventral roots (CCF = -0.91; Fig. 11J). The same experiment was repeated in four isolated preparations of the entire CNS, where FL patterns stably appeared for up to 12 min of continuous stimulations (intensity = 0.5–4.5 mA, duration* =* 1–5 ms, frequency = 1–2 Hz). Locomotor-like events were characterized by stable discharges (frequency CV = 0.14 ± 0.12, Fig. 11K) with a frequency of 0.35 ± 0.09 Hz (mean cycle period, Fig. 11K) and double alternating between pairs of VRs (homolateral CCF =  − 0.88 ± 0.06 and homosegmental CCF = -0.85 ± 0.06, Fig. 11K).

Collectively, FL patterns are evoked in the whole CNS in vitro by trains of electrical pulses applied either to lumbosacral afferents or to the VLM, proving this isolated preparation as a suitable model to study spinal circuits for locomotion in a more intact environment.

## Discussion

In the present study, we introduce a more intact in vitro preparation of the entire CNS to explore the development of brain centers and their influence on both brainstem and spinal microcircuits, which express the rhythmic activities of breathing and locomotion, respectively.

The preparation maintains the conduction of descending and ascending input along the cord and shows a fictive respiratory rhythm that remains stable for over 4 hours. Both the well-preserved tissue oxygenation in the brainstem and cortical surfaces and optimal cell viability in the internal brain structures under the surface of the ventrolateral prefrontal cortex demonstrate the reliability of the in vitro brain for the entire duration of experiments.

Collectively, data indicate that suprapontine structures affect distinct features of fictive respiration, even when legs are kept attached to explore how afferent input from the periphery tune respiration. The lack of suprapontine structures also deprives lumbar circuits of some modulatory influences, since disconnection from higher centers generates poorer motor-evoked responses. Furthermore, in the whole CNS, electrically evoked FL patterns slightly increase the total duration and coordination of cycles.

Collectively, this more intact experimental setting allows for clarifying the rostral modulation of brainstem networks and for studying supraspinal influences on spinal microcircuits.

### Influences of Suprapontine Structures on Brainstem Neuronal Networks for Respiration

Many studies about the suprapontine control of breathing indicate that multiple brain structures are involved in modulating respiration (Horn and Waldrop 1998; Fukushi et al. 2019).

In particular, the posterior hypothalamic area has been found crucial for modulating respiration in cats and rodents, hence suggesting that several neurogenic breathing disorders in humans can be ascribed to dysfunctions of the hypothalamus (Fukushi et al. 2019).

The posterior hypothalamus modulates respiratory changes during distinct emotional and arousal states and also receives input from the motor cortex (Fukushi et al. 2019). Many of the structures in the cortex and subcortex that are traditionally related to motor functions are also activated during ventilatory challenges. Cortical circuits not only receive afferent input from respiratory nuclei, but also strongly influence the respiratory control through descending projections. Indeed, phrenic and thoracic motoneurons receive descending input from the motor cortex (Rikard-Bell et al. 1985) and from the prefrontal cortex. Cortical input also reaches the midbrain through periaqueductal gray neurons, which modulate respiration when electrically stimulated (Beitz 1982). As a result, PET scans show premotor and motor cortices being active during both volitional breathing in human subjects (Colebatch et al. 1991) and during forced inspiration (Fink et al. 1996). In the latter case, basal ganglia activation was also observed (Fink et al. 1996). Starting from the pioneer studies by Spencer (Spencer 1894), electrical stimulation has been used in most areas of the cortex, decreasing respiratory frequency. Moreover, results obtained by comparing changes in the respiratory frequency during hypoxia in decerebrated or decorticated awake cats indicate that descending influences from the cerebrum inhibit the activity of medullary respiratory centers, while inputs from the diencephalon facilitate it (Tenney and Ou 1977). Accordingly, in our experiments, precollicular decerebration speeded up fictive respiration and reduced the duration of each burst. The changes in the frequency of fictive respiration after diencephalic, mesencephalic, or pontobulbar transections demonstrate that, although neonatal rat’s neuronal networks are still immature, the brain modulates the respiratory rhythm already at birth (Okada et al. 1993; Voituron et al. 2005). In the present work, we expanded previous observations using an entire CNS that also includes cortical structures. Furthermore, as opposed to the classical isolated brainstem plus spinal cord model (Suzue 1984), our CNS preparation maintains the cerebellum intact. Thus, it allows to explore any potential role of the cerebellum in selectively modulating respiratory functions, as cerebellar neurons in the rostral fastigial nucleus respond to both passive movement and respiratory challenges (Lutherer et al. 1989).

### Influences of Suprapontine Structures on Spinal Reflex Pathways and Locomotor Circuits

In the current study, ablation of suprapontine structures affects the extent of electrically evoked responses from both VRs and DRs. DRVRPs are the result of the recruitment of a local spinal microcircuit mainly confined at the segmental level, from dorsal afferents to ventral motor pools, reverberating input along the cord through intersegmental propriospinal connections to elicit motor responses that are derived lower and more delayed the farther they move from the stimulation site (example in Fig. 5). The contribution of higher brain centers to the localized pathway that generates spinal reflexes is not completely unexpected, as Wolpaw and collaborators have demonstrated that volitional modulation of H reflexes requires the integrity of the sensorimotor cortex and cerebellum (Wolpaw 2007; Chen et al. 2016).

On the other hand, DRDRPs correspond to the antidromic conduction of the primary afferent depolarization elicited by electrical pulses supplied to a close DR. In in vitro preparations from neonatal rats, a diffused dorsal spinal system connects all dorsal horns, producing almost simultaneous responses from all DRs. While bilateral DRDRPs are synchronized through commissural pathways running below the central canal, the strong coupling among DR-evoked potentials on the same side might be due to heterosegmental ipsilateral connections among dorsal horn networks (Taccola and Nistri 2005). In intact animals, the presynaptic inhibition process generating DRDRPs has been described under the control of tonic pathways descending from distinct structures in the cerebellum and cerebral cortex (Hagbarth and Kerr 1954). However, isolation of the sole spinal cord in vitro to study primary afferent depolarization might have underestimated the role of the brain in spinally processing afferent input at birth.

Although corticospinal tracts are still immature in the first week of life (Clarac et al. 2004), present data demonstrate that somehow, even at the neonatal stage, brain input already influences spinal responses. How cortical input reaches spinal targets at this stage of development, though, is debatable. Considering the immaturity of corticospinal tracts in newborns, as defined in our study by the inability to record any motor-evoked potentials in response to electrical stimulation of the surface of the cortex, it is possible that subthreshold brain input passes through deep local connections relaying to the midbrain and then downstream. We speculate that this input likely reverberates to ventral motor pools through the propriospinal dorsal network involved in the presynaptic inhibition of afferent input. Rerouting through dorsal networks may be reminiscent of the dorsoventral gradient of maturation in the connections between cortex and spinal cord during postnatal development (Lakke 1997; Martin 2005).

In our study, ablation of suprapontine structures likely removed any descending modulatory tone on evoked motor responses and was accompanied by changes in antidromic dorsal root discharges. Interestingly, in the spinal cord isolated within the first few days after birth, electrical stimulation of pathways running in the ventral funiculus modulates synaptic transmission from primary afferents to lumbar motoneurons through the recruitment of spinal interneurons that mediate presynaptic inhibition (Vinay and Clarac 1999).

In the isolated CNS preparation, the contribution of the cerebellum should also be considered, as it may facilitate the descending system that tunes spinal dorsal horn activity by exploiting the extensive connections between the cerebellum and the cerebral cortex (Hagains et al. 2011).

As opposed to the DRVRPS and DRDRPS described above, brain disconnection only produces slight changes in the episodes of locomotor-like oscillations evoked by trains of pulses applied to a DR. The same observation occurred with FL patterns recorded in vivo, regardless of whether they were recorded from intact anesthetized or decorticated cats (Millhorn et al. 1987). Moreover, the fictive locomotor patterns elicited by sacrocaudal stimulation after spinal cord transection were very similar to the actual muscle recruitment occurring during real locomotion in intact cats (Frigon 2012). More strikingly, in an in vivo preparation of a decerebrate adult mouse, spinalization did not affect the overall stable FL induced by L-DOPA plus 5HT, apart from marginal increases in rhythm frequency only (Meehan et al. 2012).

This evidence, as well as our results, is consistent with the fact that FL originates from neuronal networks located in the lumbosacral cord (Cazalets et al. 1995; Kjaerulff and Kiehn 1996; Cowley and Schmidt 1997; Kremer and Lev-Tov 1997). Yet, the small but significant changes we reported after brain disconnection, as for the total duration of FL episodes, mean cumulative depolarization peak, and flexor–extensor alternation, justify the adoption of our isolated CNS to investigate the subtle modulatory tone provided by descending input that reaches rhythmogenic spinal networks at birth.

### Motor-Evoked Responses Evoked by Electrical Stimulation

In the current study, peripheral and brainstem stimulations were used to demonstrate the presence of functional ascending and descending pathways along the cord. Electrical pulses delivered to spinal DRs successfully induced motor-evoked potentials (MEPs), as well as broader pulses applied to different spots of the pons and medulla, as already shown in the isolated CNS of opossum (Nicholls et al. 1990). These results are consistent with the development of spinal tracts. Indeed, all ascending pathways reach their targets before the rat is born: thalamocortical at E15, spinocerebellar at E17, primary afferent fibers in the gracile nucleus in the medulla oblongata at E18-21, the first spinothalamic fibers at E18, and spinothalamic and medial lemniscal at E19. Similarly, around birth, also lumbar segments are reached by descending spinal pathways, as GABAergic, serotonergic, noradrenergic reticulospinal and vestibulospinal tracts, along with rubrospinal and parafascicularis prerubralis fibers (Kudo et al. 1993; Lakke 1997; Clarac et al. 2004). However, distinct descending tracts from the hypothalamus reach lumbar spinal segments only after birth, like the fibers from the nucleus paraventricularis and area lateralis at P1, or from the zona incerta at P2 (Lakke 1997). Even slower is the maturation of the corticospinal tract, which extends to cervical and thoracic segments at P3 and, only at P6, to lumbar segments (Clarac et al. 2004). Then, corticospinal axons increase consistently until P8-10, when structurally immature axons are gradually eliminated up to the end of the second week (Joosten et al. 1987; Schreyer and Jones 1988).

Accordingly, in our experiments, there were no chances of inducing any MEPs or to record sensory-evoked potentials (SEPs) at any of the ages explored (0–3 days old), even by lowering the stimulating electrode all the way to the surface of the midbrain. Indeed, peripheral electrical stimulation has been tested for inducing cortical surface potentials only starting from P3-5 rats (An et al. 2014). The lack of MEPs or SEPs in the first days of life may be a consequence of immature cortical dendritic and axonal morphogenesis, as well as of the lodgment of synapses in the neocortex reaching circuit refinement only at P10 (Lim et al. 2018). Moreover, the correct soma-dendritic polarity of cortical neurons is not appropriately defined until 5 postnatal days (Kasper et al. 1994).

As for functional maturation, neocortical pyramidal neurons already trigger action potentials at birth (McCormick and Prince 1987), although their biophysical membrane properties are still vestigial. Indeed, only from the beginning of the second week do they differentiate into adapting and non-adapting regular spiking cells (Franceschetti et al. 1998). Then, from the second week, their action potentials grow in amplitude and shorten in duration with higher firing frequency, until completely developed by the third week after birth (McCormick and Prince 1987).

Furthermore, spinal responses to brain stimuli might also be affected by the incomplete myelinization of descending pathways. Indeed, at one day of age, immunoreactivity to myelin was detected in the lower brain stem, whereas it was absent in the rest of the brain (Bjelke and Seiger 1989). Then, during the first and second postnatal weeks, myelination continues in caudal rostral progression, spreading from the spinal cord to the medulla oblongata, pons, mesencephalon, and finally telencephalon, eventually completing during the third week after birth (Bjelke and Seiger 1989; Doretto et al. 2011; Downes and Mullins 2014).

In line with the morphological and functional development of the cortex and corticospinal pathways, pups switch from crawling to walking only at P10. A mature motor behavior is then reached only at P15 (Clarac et al. 2004), although the cortex is still evolving with the further appearance of motor maps at day 35 and its continuous enlargement until adult size around day 60 (Young et al. 2012).

### Histological and Oximetric Assessments of Brain Maintenance In Vitro

In our preparation of the entire CNS in vitro from neonatal rats, suprapontine structures already appeared to have functional links to brainstem and spinal circuits downstream, as they modulated fictive respiration and lumbar reflex activity. We speculate that, at birth, more inner brain centers, as hypothalamic areas or basal ganglia, that were inaccessible to our surface electrodes, might relay brain control over neuronal networks downstream.

Indeed, despite the absence of cortical potentials and cortically evoked spinal motor responses, histological analysis revealed the lack of astrogliosis and optimal neuron survival in the internal brain structures under the surface of the ventrolateral prefrontal cortex. Surprisingly, albeit neuronal preservation, we reported a reduced labeling of the brain-specific astroglial protein GFAP after 4 h of maintenance in vitro, especially in older rats. This evidence is reminiscent of the reduction in GFAP without any hypothalamic cell loss that has been correlated to the initial stages of hypoglycemia (Holmes et al. 2016). These findings corroborate the use of the entire CNS preparation for up to 4 h with good tissue preservation. Furthermore, it may suggest that any use of this preparation that considers longer maintenance in vitro should be accompanied by additional cell viability assays also with a detailed study of astrocyte morphology.

Interestingly, oximetric assessments indicate optimal oxygenation of the cortex after 4 h in vitro, although we do not know whether these PO_2_ levels correspond to full network functionality. However, the oxygenation we assessed in the cortex was almost twice the one we measured in the brainstem when a stable respiratory rhythm was recorded for the entire duration of the experiment. Notably, we found that PO_2_ levels in the brainstem were similar to values already reported in the literature (Okada et al. 1993; Wilson et al. 2003; Zimmer et al. 2020). Furthermore, in our settings, PO_2_ measurements on the surface of the recording chamber, close to the cortex, indicated values that were higher than those taken underneath, where the brainstem was positioned. In the current study, oxygenation within deep forebrain structures was not fully assessed. Therefore, one must adopt caution in extending the oximetric measures obtained from the superficial cortex also to inner brain areas, due to both the different metabolic requirements of distinct groups of neurons and their selective vulnerability to the lack of oxygen. Many factors contribute to the evolution of this pattern, including the development of the cerebral vasculature and the selective cellular oxidative metabolism (Ferriero 2001; McQuillen et al. 2003). However, since cortical neurons are more sensitive to hypoxia in comparison with basal ganglia and thalamus (Northington et al. 2001), we can infer that, in our preparation, inner brain structures received enough oxygen to maintain their functionality for the entire length of experiments.

The absence of cell death and astrogliosis in the brain, as well as the preserved levels of tissue oxygen throughout the experiments, support the use of the entire CNS preparation from neonatal rats to explore the development of suprapontine structures and their role in modulating brainstem and spinal circuits.

### Perspectives

The entire isolated CNS allows to study the maturation of corticospinal tracts and the developmental changes in functional coupling among cortical, brainstem, and spinal networks. Moreover, the same approach can be replicated on transgenic mice, exploiting optogenetic and epigenetic techniques for the selective activation of distinct neuronal pools regulating intrinsic rhythmic functions. We repute that the proposed experimental tool can launch new studies on the quite unexplored field of corticomotor plasticity using in vitro preparations.

## Data Availability

The datasets generated during and/or analyzed during the current study are available from the corresponding author upon reasonable request.

## Abbreviations

ANOVA: Analysis of variance
AU: Arbitrary units
CCF: Cross-correlation function
C: Cervical
CNS: Central nervous system
DAPI: 4', 6-Diamidino-2-phenylindole
DR: Dorsal root
DRDRP: Dorsal root dorsal root potential
DRVRP: Dorsal root ventral root potential
GFAP: Glial fibrillary acidic protein
l: Left
L: Lumbar
P: Postnatal
PBS: Phosphate-buffered saline
r: Right
Thr: Threshold
T: Thoracic
VR: Ventral root
